# Mechanistic Evidence of *Andrographis paniculata* (Burm. f.) Wall. ex Nees, *Pelargonium sidoides* DC., *Echinacea* Species and a Combination of *Hedera helix* L., *Primula veris* L./*Primula elatior* L. and *Thymus vulgaris* L./*Thymus zygis* L. in the Treatment of Acute, Uncomplicated Respiratory Tract Infections: A Systematic Literature Review and Expert Interviews

**DOI:** 10.3390/ph16091206

**Published:** 2023-08-24

**Authors:** Liesbeth B. M. Veldman, Eefje Belt-Van Zoen, Erik W. Baars

**Affiliations:** 1 Faculty of Healthcare, University of Applied Sciences Leiden, 2333 Leiden, The Netherlands; 2 Louis Bolk Institute, 3981 Bunnik, The Netherlands

**Keywords:** antimicrobial resistance, mechanistic evidence, respiratory tract infections, multi-targeted

## Abstract

Reducing inappropriate antibiotic (AB) use by using effective non-antibiotic treatments is one strategy to prevent and reduce antimicrobial resistance (AMR). *Andrographis paniculata* (Burm. f.) Wall. ex Nees, *Pelargonium sidoides* DC., *Echinacea* species and a combination of ivy (*Hedera helix* L.), primrose (*Primula veris* L./*Primula elatior* L.) and thyme (*Thymus vulgaris* L./*Thymus zygis* L.) have promising clinical effects in uncomplicated, acute upper respiratory tract infections (URTI) treatment. However, mechanistic evidence of these herbal treatments is lacking. The objective of this Pstudy is to provide an overview of mechanistic evidence for these effects. Thirty-eight databases were searched. Included studies were mechanistic studies (in vitro, animal, and human studies and reviews) on these herbs; published before June 2021. Non-mechanistic studies or studies on combinations of herbs other than ivy/primrose/thyme were excluded. Furthermore, three experts in traditional, complementary and integrative healthcare (TCIH) research and pharmacognosy were interviewed to collect additional expert knowledge. The results show that *A. paniculata* acts through immunomodulation and antiviral activity, possibly supplemented by antibacterial and antipyretic effects. *P. sidoides* acts through antiviral, indirect antibacterial, immunomodulatory and expectorant effects. *Echinacea* species likely act through immunomodulation. The combination of ivy/primrose/thyme combines secretolytic and spasmolytic effects from ivy with antibacterial effects from thyme. Studies on primrose were lacking. This mechanistic evidence supports the difference-making evidence from clinical studies, contributes to evidence-based recommendations for their use in URTI treatment, and guides future mechanistic studies on URTI treatments.

## 1. Introduction

Antimicrobial resistance (AMR) is a worldwide leading public health problem affecting the lives of millions of people. In 2019, an estimated 4.95 million deaths were associated with bacterial AMR [1]. Policies at global, regional, and national levels focus on six main strategies to reduce antibiotic use. These strategies are (1) preventing infection and controlling resistant bacteria, (2) monitoring both infection prevention and control of resistant bacteria, (3) researching antibiotic resistance and antibiotic use, (4) only using antibiotics when appropriate, (5) reducing the use of antibiotics, and (6) developing new antibiotics [2]. However, in the European Union and European Economic Area over the period of 2010–2019 the average total AB consumption reduced only by 0.4% in the community and hospital sector combined. In each of these sectors alone the decrease was not statistically significant [3]. Finding ways to further, and more effectively, lower antibiotic prescription and consumption, is, therefore, of great importance.

URTIs are a leading cause of inappropriate use of antibiotics, especially in children [4]. In case of a bacterial pathogen, this often involves *Streptococcus pyogenes*, *Streptococcus pneumoniae*, or *Haemophilus influenzae* [5]. However, the vast majority of URTIs (an estimated 70%) are caused by viruses such as rhinoviruses, coronaviruses, influenzaviruses A or B, or respiratory syncytial viruses [6,7,8]. It is not surprising that antibiotics often only have small or negligible symptomatic benefit for URTI patients and are not included in most treatment guidelines [9]. Even though many URTIs are self-limiting, the patients’ need for symptom relief remains. Therefore, non-antibiotic treatment strategies that help treat symptoms are required, especially in non-bacterial and non-severe cases.

Currently, traditional, complementary and integrative healthcare (TCIH) is not yet included as a strategy to counter AMR. However, it could prove to be of great value as a strategy for both reducing antibiotic consumption and promoting the appropriate use of antibiotics, as was observed in several observational studies. For example, a retrospective cross-sectional analysis of national primary care in England found that health centres that employed general practitioners (GPs) trained in TCIH or integrative medicine (IM) prescribed fewer antibiotics (relative risk = 0.78, 95% confidence interval (CI): 0.64 to 0.97) [10]. Furthermore, French patients who consulted a GP additionally trained in homeopathy used significantly fewer antibiotics for uncomplicated acute respiratory tract infections (URTIs) (OR = 0.43, 95% CI: 0.27–0.68) [11]. In children treated by anthroposophic GPs for URTIs and ear infections, significantly fewer antibiotics were prescribed as well (OR for non-prescription = 6.58, 95% CI: 3.45–12.56). In addition, there was a trend to quicker symptom relief and a higher caregiver satisfaction with anthroposophic GP treatment compared to conventional GP treatment [12]. In addition to reducing the use of antibiotics, TCIH treatments can contribute to reducing AMR development by offering new approaches to treat and prevent microbial infections such as URTIs.

TCIH treatments hold a unique potential to aid in the reduction in antibiotic use as these treatments approach the problem of URTI in a different way. Most conventional treatment strategies focus on creating specific immunity and fighting the microorganisms. In TCIH strategies, on the other hand, the main focus of treatment is on enhancing the individual’s resilience; improving the general physiological ability to self-manage and adapt to infections by strengthening the individual’s self-regulating abilities [13]. Resilience is a holistic concept that refers to an individual’s ability to recover from or optimise the functioning in disease or disability [14]. Many resilience-enhancing TCIH treatment strategies are promising, but lack high-quality scientific evidence [13]. Therefore, more research is needed into their (cost)effectiveness.

### 1.1. Clinical Evidence for TCIH Treatments in URTI

The efficacy of some natural medicinal products for treating acute, uncomplicated URTIs has been shown in moderate- to high-quality clinical studies. Systematic reviews on their efficacy show promising effects. A recent meta-analysis of meta-analyses identified the four most promising TCIH treatments for RTIs [15]. These are *Andrographis paniculata* (Burm. f.) Wall. ex Nees and *Pelargonium sidiodes* DC. for both general and specific URTI symptoms (e.g., cough and sore throat) [16,17,18,19,20,21], *Echinacea* spp. for common cold [20], and a combination of ivy (*Hedera helix* L.), primrose (*Primula veris* L./*Primula elatior* L.), and thyme (*Thymus vulgaris* L./*Thymus zygis* L.) for cough only [22,23].

*Andrographis paniculata* is an extremely bitter herb, of which the aerial parts—and sometimes the roots—are used in Ayurveda, Siddha, homeopathy, naturopathy, and traditional Chinese and Thai medicine for various indications [24,25]. A systematic review and meta-analysis of clinical trials showed that *A. paniculata* significantly improved overall symptoms of acute URTI. Specifically, it improved cough (*n* = 596, standardised mean difference (SMD): −0.39, 95% CI: −0.67, −0.10; *p* = 0.008) and sore throat (*n* = 314, SMD: −1.13, 95% CI: −1.37, −0.89; *p* < 0.00001) and significantly shortened the duration of these symptoms when compared to standard treatment. In addition, no major adverse effects were found [17]. These findings were confirmed in another systematic review and meta-analysis which found *A. paniculata* significantly improved cough in URTI and common cold (SMD = −1.00, 95% CI: −1.85, −0.15; *p* < 0.001) [20].

*Pelargonium sidoides* is a medicinal plant from South Africa which belongs to the same family as the ornamental geraniums (*Geraniaceae*) [26]. In the 19th century, it was brought to Europe, where a standardised ethanolic extract of it was made, called EPs 7630 or Umckaloabo, which has been researched extensively. In a meta-analysis of clinical trials, treatment with EPs 7630 in adult acute bronchitis patients significantly reduced the bronchitis severity score (WMD 2.80 points, 95% CI: 2.44–3.15) [18]. Furthermore, in children under 18, EPs 7630 has been found to be effective in treating URTI (responder rates: RR = 2.56; 95% CI: 1.54–4.26; *p* < 0.01) and to be safe (patients with adverse events: RR, 1.06; 95% CI: 0.42–2.66; *p* = 0.9) [16]. Even in young children, under 6 years old, similar findings were reported [19].

*Echinacea* is a genus that includes nine different plants, of which three species, *E. purpurea* L. Moench, *E. angustifolia* (DC.) Hell., and *E. pallida* (Nutt.) Nutt., are most often used for medicinal purposes. The most used species is *Echinacea purpurea*. This species occurs in about 80% of all commercially available products containing some Echinacea species [27]. The evidence from clinical trials on *Echinacea’*s effectiveness in treating URTIs is contradictory. A systematic review of clinical trials found limited evidence for *Echinacea* reducing cough as a symptom of URTI and the common cold in adult patients (SMD = −0.68; 95% CI: −1.32, −0.04; *p* = 0.04) [20]. However, another systematic review found no effect of *Echinacea* preparations in the treatment and prevention of the common cold when compared to a placebo [28].

Ivy, primrose, and thyme are three plants that are medicinally used in European phytotherapy [29,30,31]. Primrose and thyme are also used in anthroposophic medicine. There are different products on the European market that contain different combinations of these herbs such as Bronchipret, Hedelix, Prospan, and Mintetten. A meta-analysis of clinical trials on the treatment with ivy, primrose, and thyme combinations found strong evidence for these to be effective in the treatment of cough in URTI and the common cold (RR = 1.40, 95% CI: 1.23–1.60; *p* < 0.001) [20].

### 1.2. Study Aims

In short, for each of these treatments there are clinical data that provide evidence of their effects in URTI treatment. Exploring the underlying mechanisms of these clinically observed effects could help when working towards evidence-based recommendations on the practical applications of these treatments. The underlying mechanisms might explain the unique potential of TCIH treatments to enhance a person’s resilience [13]. Hence, the main aim of this study was to further investigate the working mechanisms that underlie the effects of these four treatment strategies.

### 1.3. Research Questions

The main research question was: what are the underlying mechanisms by which *Andrographis paniculata*, *Pelargonium sidoides*, *Echinacea* spp., and ivy/primrose/thyme positively influence acute and uncomplicated URTI symptoms?

The research hypothesis was that all of these plants decrease URTI symptoms through a combination of direct antibacterial and antiviral effects, as well as through an enhancement in the host’s resilience [13]. In line with this hypothesis, sub-research questions were:

1. What are the direct antibacterial and antiviral effects of each treatment?
2. What are the effects on the immune system of each of these treatments?
3. What are the effects on expectoration and disease management of each of these herbs?
4. Are there effects resulting only from the combination of at least two of ivy, primrose, and thyme?

## 2. Materials and Methods

The PRISMA guidelines were followed. The study protocol was not registered in an online database.

### 2.1. Search Strategy

In total, three searches were executed in 38 databases. The first search was aimed at obtaining a broad picture of the available mechanistic evidence for the selected herbs in URTI treatment. To limit the number of results, only review articles were searched for. The concept of the working mechanism was further specified to effects on (1) bacterial growth, (2) viral replication, (3) immune (cell) function, or (4) URTI disease management.

If for a specific herb no review or only one review was found, a second literature search was performed. This second search was performed to ensure that for each herb a sufficient number of studies were included to obtain a good picture of the available evidence and to allow for the potential discovery of new insights. In this second search, all types of mechanistic studies, not only review articles but also mechanistic clinical trials, animal studies, and in vitro studies, were included.

Furthermore, if the most recent review that was retrieved in the first search was more than 5 years old at the time at which the initial search was performed (<2016), an additional search was performed. This search was performed to ensure that the present study contains recent developments and included all types of mechanistic studies that were published after the most recent review retrieved.

#### 2.1.1. Search Terms

A qualified information specialist (University of Applied Sciences Leiden) developed a list of appropriate search terms for the electronic databases (search terms provided in Appendix B Search Terms). These search terms consisted of (1) various types of URTIs, (2) various alternative names for the different plants and main active compounds, and (3) different terms describing working mechanisms and the three main proposed working mechanisms (direct antibacterial/antiviral effects, immunomodulatory effects, and expectorant effects). An additional search term ‘“systematic review” OR “meta-analysis” OR “review” was included to narrow down the results to review articles for the first search. The exact search terms that were used can be found in Appendix B Search Terms.

#### 2.1.2. Sources

The following databases were searched: Academic Search Ultimate, Beeld en Geluid op school, Business Source Premier, Chemische Feitelijkheden, CINAHL with full text, Chromedia, Cochrane Library, DART-Europe, Databank Vaktherapie, Delpher, Education Research Complete, ERIC, GreenFILE, HBO Kennisbank, JAR (OpMaat), Kennisbank Diversiteit en Emancipatie, Kluwer Navigator, Legal Intelligence, Library, Information Science & Technology Abstracts, MEDLINE with full text, NARCIS, National Guideline Clearinghouse, Nexis Uni, PEDro, Picarta, Psychology and Behavioral Sciences Collection, PsycINFO, PubMed, Regional Business News, SAGE journals, Schooltv, ScienceDirect, Sociaal Digitaal, SpringerLink, Teacher Reference Center, Tijdschrift voor HRM, TRIP database, and Wiley Online Library.

Additionally, the bibliographies of selected studies were searched for relevant references, ESCOP monographs were consulted, and pharmaceutical companies that produce products with the selected herbs were asked for studies on their mechanism of action.

### 2.2. Eligibility

#### 2.2.1. Inclusion Criteria

Inclusion criteria required that the studies evaluated the working mechanism in the treatment of URTI of the herbs *A. paniculata*, *P. sidoides*, *Echinacea* species, ivy, primrose, or thyme or of active compounds present in these herbs. Only combinations with standard antibiotics and combinations of ivy, primrose, and thyme were included, as the effects of these combinations are relevant to treatment of URTI and for integrating the TCIH treatments in clinical practice. Studies on effects in the lungs were included as these effects could plausibly be translated to infections of the upper respiratory tract.

#### 2.2.2. Exclusion Criteria

Studies reporting only on a combination of one of the six studied herbs with other herbs were excluded as effects could not with certainty be attributed to the studied herb. Other exclusion criteria concerned studies reporting only on mechanisms that are unrelated to URTI treatment, as they were not relevant to the research question that is focussed on the treatment of URTIs. Clinical trials that did not investigate the mechanism of antiviral, antibacterial, or immunomodulatory effects were excluded. Articles that were not scientific, e.g., popular magazine articles were excluded, as these did not offer reliable information on working mechanisms and would, therefore, not contribute to the methodological quality of this study. Articles written in languages other than English, German, or Dutch were excluded as the authors were not able to read and understand these. In the third search, articles that were published before the most recent review retrieved were excluded. A list of excluded studies with the reason for exclusion is provided as a Supplementary File (Table S1: Study selection).

### 2.3. Data Selection and Extraction

All identified studies were collected in an Endnote file. First, duplicates were removed, and then, a selection was performed on title, abstract, and full text. The results of the selection were recorded in an Excel file. Finally, the full texts of the selected studies were read and relevant data were recorded in a standard extraction file. The selection was performed by two authors independently (LV and EBZ). The results of this were compared and differences were discussed until an agreement was reached. If needed, a third author was consulted to resolve differences. Data extraction was performed by two authors independently (LV and EBA). The results of this were compared and differences were discussed until an agreement was reached. If needed, a third author was consulted to resolve differences.

### 2.4. Interviews

In order to integrate experiential knowledge and compare this to the findings from the literature, interviews with experts in the field were also conducted. For this purpose, several academic researchers connected to the EUROCAM or AZKIM network were approached and asked to participate in an online interview. From these, a medical doctor and professor of medicine with broad clinical experience in internal medicine, gastroenterology, and complementary medicine (Prof. Dr. Med. Roman Huber, Universitätklinikum Freiburg), as well as an Emeritus Professor of pharmacognosy (Emeritus Prof. Dr. Liselotte Krenn, University of Vienna) and a pharmacist specialising in herbal products (Dr. Mathias Schmidt, Herbresearch Germany) shared their experience. The topics discussed included the working mechanisms of the investigated herbs and more general methodological questions on mechanistic research. The interviews were semi-structured and were performed in an online (Microsoft Teams) or telephone meeting. When they were performed through an online meeting they were recorded. Notes were taken and the main messages are presented in the report.

### 2.5. Analysis

For each herb, findings from the literature and interviews were grouped together into relevant themes (antibacterial, antiviral, immunomodulatory, and other effects). No risk of bias assessment was performed. Specific information on the mechanism of action of individual active compounds was described when retrieved. If such detail in description is lacking, such information was not available in the included studies.

## 3. Results

### 3.1. Search Results

**Figure 1:**
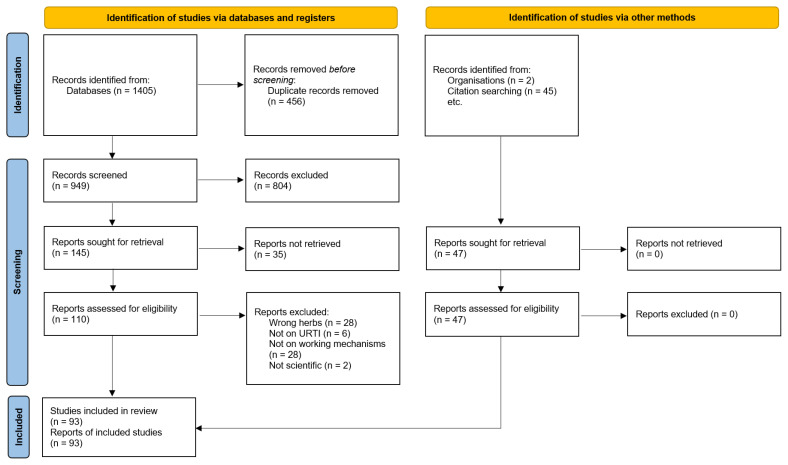
PRISMA flow diagram showing the identification and screening of studies retrieved.

Three systematic searches were performed and yielded a total of 1405 reports. This included 996 articles from the first search, that included only review articles for all herbs; 319 articles from the second, that included all types of mechanistic studies on herbs for which only one or no reviews were retrieved during the first search; and another 120 articles from the third search, that included all types of mechanistic studies and that was performed if the most recent review article retrieved was more than 5 years old. After the screening of title, abstract, and full-text, 46 reports were chosen for data extraction. From the first search, 17 reviews were included. As this yielded only one review on thyme [32] and none on ivy or primrose, a second search for these herbs was performed, which yielded 21 additional studies. Only for *P. sidoides* was the most recent review more than five years old [26], hence, an additional search for more recent studies was performed for this herb. This yielded an additional eight studies. A PRISMA flow diagram of the selection and inclusion process is given in Figure 1. Another 45 studies were added after searching the bibliographies of the selected studies. Two more studies were included after consulting pharmaceutical companies, yielding a total of 93 studies for full review. A total of 17 of these 93 studies were reviews, 6 were mechanistic clinical trials, 10 were animal studies, and 60 were in vitro studies. A table describing the study characteristics can be found in Appendix A Study Characteristics.

### 3.2. Andrographis paniculata (Burm. F) Nees

Of *A. paniculata* different effects are known, including antibacterial, antiviral, immunomodulatory and antipyretic effects. An overview of these is given in Table 1. Below they are discussed in further detail.

#### 3.2.1. Antibacterial Effects

Several studies have found a direct antibacterial effect of *A. paniculata* and some of its compounds. For example, a methanolic extract of the leaves of *A. paniculata* showed a significant inhibition of *S. aureus*, *E. coli*, *S. typhimurium*, and *B. subtilis* in an agar well diffusion assay [24,33]. Furthermore, antibacterial effects against methicillin-resistant *S. aureus* (MRSA) and *E. faecalis* were found. When different solvents were tested, a methanol extract was more potent compared to water and hexane extracts [34]. More specifically, these effects were thought to be caused by the terpenoids, flavonoids, or phenolic acids present in *A. paniculata* [24].

The individual compound andrographolide inhibited *Staphylococcus aureus* with a very high MIC of 100 µg/mL. Reductions in DNA synthesis (31%), RNA synthesis (26%), and protein synthesis (36%) were found which were comparable to the antibiotic ciprofloxacin (with 25% incorporation). Furthermore, andrographolide affected the quorum sensing system (QSS), which resulted in a reduced production of extracellular polymeric substance and inhibited virulence factors [35].

However, when comparing the ethanolic extract of *A. paniculata* to andrographolide alone, the ethanolic extract showed a stronger inhibitory effect with *E. coli*, *K. pneumoniae*, and *P. vulgaris*. Only the inhibition of *S. pneumoniae* was stronger with andrographolide alone [36]. Looking at other compounds that could be involved in the antibacterial effect, the noriridoides andrographidoides A–E were investigated as well. These are mostly present in the roots of *A. paniculata*. However, they did not show any antibacterial effect on *E. coli*, *S. aureus*, *S. epidermidis*, *P. aeruginosa*, and *B. subtilis* (MICs > 100 µg/mL) [37].

##### Restoring Antibiotic Sensitivity and Reducing Biofilm

When looking closer at the inhibitory effects on bacteria, it was found that andrographolide could also help to restore the sensitivity of bacteria to antibiotics. Against *P. aeruginosa*, a direct inhibition of this kind was shown. The mechanism involved here was a reduced expression of the mexAB-oprM efflux pump, which is responsible for resistance to several antibiotics [35].

There could also be a more indirect mechanism involved. Some bacteria can form a multilayer structure called a biofilm. When pathogenic bacteria form such a structure it can form a barrier that protects them from the host’s immune system and antibiotic drugs. The formation of a biofilm is, therefore, another mechanism of reducing sensitivity to antibiotics. Some compounds present in *A. paniculata* have been shown to reduce the biofilm of certain bacteria. The compounds andrographolide, 14-deoxyandrographolide, and andrograpanin reduced the biofilms of *P. auruginosa* and *S. aureus*. With 14-deoxyandrographolide and andrograpanin, the inhibition was dose-dependent and reached 54% and 56%, respectively, at 0.15 mM concentration. Their combination with gentamicin or azithromycin enhanced the effect up to 90% and 92% inhibition, respectively. Andrographolide was less potent and inhibited biofilm production of *P. auruginosa* up to 40% alone and up to 60% in combination with another antibiotic [35].

##### Reducing Bacterial Adhesion

Finally, *A. paniculata* can reduce URTIs caused by bacteria by reducing the adhesion of pathogenic bacteria to the epithelium of the lungs and thereby reducing respiratory colonization. This has been shown for example with andrographolide. This compound has been shown to inhibit bacterial adhesion of *E. coli* and *S. epidermidis* to epithelial cells of the lungs [35].

#### 3.2.2. Antiviral Effects

Antiviral effects have also been reported. Both the water and ethanol extracts of the leaves, for example, inhibited the replication of avian influenza A virus (H5N1) in MDCK cells at non-toxic concentrations (IC50 = 38 ± 1 µg/mL). The compound 14-deoxy-11,12-dehydroandrographolide decreased the mRNA and protein expression of nucleoprotein and blocked viral ribonucleoprotein complexes from nuclei. Furthermore, this compound was shown to increase survival in mice after an infection with H5N1 to 33% at day 9, instead of 0% with no treatment [39].

A synthetic derivative of andrographolide, 14-α-lipolyl andrographolide, was tested both in vitro and in vivo. In a hemagglutinin inhibition assay an inhibition of viral absorption of both avian and human influenza A virus (H9N2, H5N1 and H1N1) into red blood cells was observed, with MICs ranging from 5.3 to 16.8 mM. The antiviral effect of *A. paniculata* was further shown in vivo. The oral administration of 14-α-lipolyl andrographolide to mice infected with either avian (H9N2 or H5N1) or human (H1N1) influenza A virus reduced death rate and prolonged life. Supplementation started 24 h before infection and was continued for seven days. 14-α-lipolyl andrographolide was the most potent and at a very high dose of 200 mg/kg bodyweight/day it increased survival to 100% in H1N1-infected and to 80% in both H9N2- and H5N1-infected mice. Andrographolide and 14-deoxy-11,12-dehydroandrographolide were also active though less potent compared to 14-α-lipolyl andrographolide. All were less potent than the control drug ribavirin, that resulted in 100% survival with each virus at a 150 mg/kg bodyweight/day dose [38].

#### 3.2.3. Immunomodulatory Effects

Several studies show that *A. paniculata* can affect the functioning and proliferation of white blood cells. For example, an ethanolic extract of *A. paniculata* was found to stimulate both specific and non-specific immunity in mice injected with sheep red blood cells. Both antibody and delayed hypersensitivity were stimulated (*p* < 0.001) as well as macrophage migration index, phagocytosis of 14C-leucine labelled *E. coli*, and proliferation of splenic lymphocytes. Moreover, andrographolide and neoandrographolide were tested and showed similar effects, though less strong when compared to the ethanol extract [47]. Furthermore, in human peripheral blood lymphocytes the dichloromethane fraction of a methanol extract of *A. paniculata* increased proliferation. This effect was observed at low concentrations, with a 52% increase in proliferation at a concentration of 2.5 µg/mL. Further fractioning identified the three diterpenes andrographolide, 14-deoxyandrographolide, and 14-deoxy-11,12-didehydroandrographolide. All three compounds alone were also found to increase the proliferation of lymphocytes [40].

Furthermore, general anti-inflammatory effects have been observed. For example, both andrographolide and dehydroandrographolide inhibited COX-1 while andrographolide and neoandrographolide inhibited the COX-2 enzyme in ionophore A23187-induced human platelets [45]. Furthermore, the ethanol extract was shown to completely prevent the formation of carrageenan-induced paw oedema in BALB-c mice, when 5 mg was given intraperitoneally for five days before the induction of the oedema [46].

##### Regulation of Macrophage Functioning

More specifically focusing on macrophage functioning, *A. paniculata* can reduce the inflammatory response in macrophages. For example, the levels of NO, IL-1β, IL-6, and PGE2 produced by J774A.1 murine macrophages in response to LPS stimulation were reduced by a methanol extract of *A. paniculata* at concentrations that were not cytotoxic (<50 µg/mL) [44]. Further investigations into the compounds involved in these effects revealed that andrographolide and isoandrographolide both reduced NO, IL-1β, IL-6, and PGE2 after LPS stimulation. Furthermore, the *A. paniculate* constituent 7-O-methylwogonin reduced NO, IL-1β, and PGE2, whilst skullcapflavone-I reduced NO, IL-6, and PGE2 [43].

Looking closer at the compound andrographolide, in in vivo experiments, this compound reduced the increase in TNF-α and IL-6 that followed infection of the lungs with *S. aureus*, while keeping them high enough to adequately manage the infection [35]. Moreover, in mice infected with H1N1, treatment with 10 mg/kg bodyweight andrographolide decreased the expression of TNF-α, IL-6, and IL-10; NFκB signalling was also decreased [39]. In in vitro experiments, andrographolide inhibited IL-1β and IL-6 as well as TNF-α and IL-10 at 10 µg/mL in human ionophore A23187-induced human platelets. Furthermore, gene expression of cytokine receptors (TNFSF14, TNF, TNFRSF6, and IL1A) and chemokines (CCL8 and CXCL11) were downregulated as well as genes of the TLR family and the JAK/STAT and NFκB signalling pathways [45].

Furthermore, andrographolide was found to inhibit macrophage responses to IL-4 or LPS and regulated the polarization of macrophage phenotype, favouring the less inflammatory type 2 macrophages. Furthermore, it downregulated the mannose receptor in response to IL-4 and major histocompatibility complexes MHC-1, CD40, CD80, and CD85 in response to LPS. In accordance with these results, specific antibody presentation was downregulated in mice receiving 1 mg/kg bodyweight andrographolide via peritoneal injection for seven days. This effect was paired with downregulation of AKT and ERK1/2 phosphorylation in the in vivo experiment, indicating that the MAPK and PI3K pathways could be involved [42].

In addition, the compound andrograpanin reduced NO, TNF-α, IL-6, and IL-12(p70) in LPS-activated macrophages dose-dependently, at doses ranging from 15 to 90 µM, by downregulating iNOS and gene expression levels of TNF-α, IL-6, and IL-12(p70). Furthermore, it was suggested that MAPK pathways are involved, as the phosphorylation of p38 MAPKs was also downregulated in a dose-dependent way. The ERK1/2 and SAPK/JNK pathways were unaffected [41].

#### 3.2.4. Antipyretic Effects

An antipyretic effect has been observed in rats with both the aqueous and chloroform extracts, when compared to petroleum ether and methanolic extracts. This was thought to be caused by the diterpenoid lactones. At doses of 400 or 800 mg/kg, the observed effect was significant [24].

### 3.3. Pelargonium sidoides (Thunb.) R. Knuth

Of *P. sidoides* different effects are known, including antibacterial, antiviral, immunomodulatory, expectorant and antitussive effects. An overview of these is given in Table 2. Below they are discussed in further detail.

#### 3.3.1. Antibacterial Effects

Different studies have investigated a possible direct antibacterial effect of *P. sidoides*. However, most of these only found a weak antibacterial effect with MICs generally starting at 97 µg/mL [26,67,68,69]. However, an indirect antibacterial effect could be caused by *P. sidoides* affecting the adherence of certain bacteria to specific tissues.

For example, changes in adhesive behaviour after exposure to EPs 7630 were observed with *S. pyogenes*. In the control group, adhesion of *S. pyogenes* to human epithelial (Hep-2) was 80%. After 120 min incubation with 30 µg/mL EPs 7630 this adhesion was reduced on average by 46%. This effect was time- and dose-dependent and occurred after pretreatment of bacteria, but not after pretreatment of HEp-2 cells. This suggested that EPs 7630 influence bacterial adhesion factors rather than host adhesion factors [48]. A further study confirmed these findings with 30 µg/mL EPs 7630 in Hep-2 cells [49].

Furthermore, when looking into the compounds that could be responsible for this effect, it was found that both the methanol insoluble and methanol soluble fraction exhibited this effect. At concentrations of 30 µg/mL, inhibition of adhesion to Hep-2 cells of 35% and 30%, respectively, was observed. Furthermore, tannin-free fractions showed no effect on adhesion, which suggested that proanthocyanidins are responsible for the anti-adhesive effect. Especially, the (epi)gallocatechin constituent flavanyl units appeared to be crucial for this effect [49].

In addition to the decreased adhesion to human Hep-2 epithelial cells, the adhesion to buccal epithelial cells was increased. This increase was about sevenfold after pre-incubation for 120 min with 30 µg/mL EPs 7630. This effect was observed both with pre-incubation of the bacteria and the buccal cells. As buccal cells are replaced rapidly, increased adhesion to these cells could prevent infectious organisms from reaching the airways. Instead they are hypothesized to be swallowed with shedding buccal cells [48].

#### 3.3.2. Antiviral Effects

Extract of *P. sidoides* (extraction method unknown) inhibited influenza A virus (H1N1 and H3N2) in MDCK cells with a mean IC50 between 7.80–11.67 µg/mL [50]. EPs 7630 also reduced virus replication of influenza virus (H1N1 and H3N2), as well as respiratory syncytial virus (HCo-229E), parainfluenza virus type 3, and coxsackievirus A9. It did not affect the virus replication of non-enveloped adenoviruses or rhinoviruses. However, *P. sidoides* did increase the survival of bronchial epithelial cells infected with rhinoviruses [51]. 

Underlying to this, *P. sidoides* appeared to interact with both the membrane of the cells and the virus envelope in order to prevent viral plaque build-up [50]. In rhinovirus-infected bronchial epithelial cells, EPs 7630 has been shown to downregulate docking proteins, such as cell surface calreticulin and inducible co-stimulator and its ligand, while simultaneously upregulating antibacterial proteins (see Section 3.3.3 Immunomodulatory Effects) [51,52]. Other membrane proteins that could interact with the virus, such as myeloid differentiation factor 88, TLR2/4, or intercellular adhesion molecule 1, remained unchanged [52]. Interaction with the cell membrane that prevented hemagglutination was also confirmed in human erythrocytes [50].

Another remarkable effect of EPs 7630 that could contribute to the antiviral effect was the upregulation of vitamin D receptors in human bronchial epithelial cells through Erk1/2 MAPK signaling. This has been observed with concentrations of EPs 7630 of 5 µg/mL or higher. Furthermore, the response of the vitamin D receptor to vitamin D was enhanced, which caused a 2.1 times stronger antiviral response against rhinovirus 16 in human bronchial epithelial cells treated with 5 µg/mL EPs 7630 [53].

#### 3.3.3. Immunomodulatory Effects

In general, there are two ways in which *P. sidoides* influences the immune functioning. First, it can reduce harmful inflammation. For example, in a rat model for acute bacterial bronchitis, EPs 7630 at doses of 30 and 60 mg/kg bodyweight reduced the degree of tracheal lesions. This effect was paired with decreased serum levels of malondialdehyde, which could indicate that this effect was at least partially mediated by an upregulation of superoxide dismutase and, consequently, a reduced level of oxidative stress [54]. Furthermore, a reduction in E-cadherin was observed in human bronchial epithelial cells exposed to 5 µg/mL EPs 7630. This could indicate a decreased thickening of the airway wall during infection or inflammation [53]. Furthermore, EPs 7630 prevented asthma attacks in children triggered by rhinovirus by decreasing the inflammation caused by increases in IL-6, IL-8, and IL-16. Similarly, EPs 7630 has been show to decrease levels of IL-6 and IL-15 in serum, while increasing levels of immunoglobulin in athletes during strenuous exercise [51].

Further proof of an anti-inflammatory effect of *P. sidoides* in infection was found with studies on sickness behaviour. In response to infection, proinflammatory cytokines are produced which induce sickness behaviour. This includes anorexia, depressed activity, listlessness, and malaise. In male NMRI mice, sickness behaviour was induced by intraperitoneal injection with 400 µg/kg bodyweight LPS. Oral administration of a standardised *P. sidoides* extract (Umckaloabo) at doses ranging from 100 to 400 mg/kg bodyweight one hour before LPS administration dose-dependently antagonised reduced activity and listlessness [55]. Furthermore, administration of 100–400 mg/kg bodyweight EPs 7630 one hour before LPS injection caused this effect in mice. Further investigation into different molecular weight fractions showed that the high molecular weight fraction (>30 kDa) was responsible for this effect [56].

Second, *P. sidoides* can increase the immune response by enhancing phagocytic activity, oxidative burst, intracellular killing of pathogens, and the release of NO in macrophages, as well as inducing the release of TNF-α, IL-1, and IL-12 [67]. EPs 7630 has also been shown to increase levels of antimicrobial proteins in host cells. Below, these immune-stimulating effects will be discussed one by one in greater detail.

##### Effects on Macrophages

First, the enhancement in phagocytotic activity and oxidative burst was shown with EPs 7630. This extract increased the number of phagocytosing human peripheral blood phagocytes in a dose-dependent manner with concentrations between 1 and 30 µg/mL after 2–10 min exposure. The maximal increase was 56% after 2 min exposure to 30 µg/mL EPs 7630 (*p* = 0.002). Furthermore, the number of burst-active phagocytes increased dose-dependently at all timepoints between 2 and 30 min. The maximal increase in this was 120% after 4 min of exposure to 30 µg/mL [58].

In addition, the enhanced intracellular killing of pathogens was shown in the same experiment. *C. albicans* was used as the pathogen and the number of surviving *C. albicans* cells decreased significantly with 30 µg/mL EPs 7630, though not with lower concentrations. The highest reduction in viable yeast cells was 31% and was observed after 120 min incubation with 30 µg/mL [58]. In another experiment, it was shown that the methanol, petrol ether, ethyl acetate, and n-Butanol extracts of *P. sidoides* reduced the intracellular survival of *L. donovania* in murine macrophages. The EC50 concentration ranged from 0.1 to 3.3 µg/mL. Furthermore, gallic acid and methyl esters found in *P. sidoides* reduced the survival of L. donocania, with EC50’s of 4.4 µg/mL and 12.5 µg/mL, respectively [59]. Moreover, when murine macrophages were infected with L. monocytogenes, EPs 7630 increased the intracellular killing dose-dependently at concentrations between 1 and 30 µg/mL. This effect was enhanced by IFN-γ, and when 10 µg/mL EPs 7630 was combined with 100 U/mL IFN-γ the effect was almost equal to 10 µg/mL LPS with 100 U/mL IFN-γ [60].

Finally, the production of NO by murine macrophages infected with *L. monocytogenes* was increased when exposed to 30 µg/mL EPs 7630 [60]. Similarly, iNOS expression increased in murine macrophages infected with *L. donovania* and exposed to EPs 7630. However, blocking iNOS did not affect the increased intracellular killing of *L. donovania* [59].

##### Cytokine Production and Effects on Other Immune Cells

In human blood immune cells, EPs 7630 increased TNF-α, IL-6, and IL-10 dose-dependently [63], and in macrophages, pro-inflammatory cytokines such as TNF-α, IL-1α, IL-1β, and IL-12 increased [60]. In RAW 264.7 cells, treatment with 50 µg/mL EPs 7630 increased the mRNA levels of IL-1, IL-12, IL-18, TNF-α, IFN-α, and IFN-γ. This was observed in uninfected cells, but the increase was stronger in cells infected with L. major. Further investigation found that this effect occurred with the methanol-soluble fraction, but not with the methanol-insoluble fraction [64]. Further explanation might involve MAPK activation, which was found with EPs 7630 treatment in human monocytes, while p38 was strongly inhibited [63].

Furthermore, the interleukins 17 and 22 increased with EPs 7630 treatment of human memory T-cells. Especially, IL-22 increased strongly. Direct contact of the T-cell with EPs 7630 was necessary, as well as soluble mediators produced by monocytes upon EPs 7630 treatment [61]. Stimulation of T-cells with EPs 7630 alone did not produce such an effect [61,63]. Interestingly, this IL-22 was then found to increase the release of antimicrobial protein S100A9 in lung epithelial cells and pulmonary tissues [61].

In mechanistic clinical trials, there have also been changes in cytokine and chemokine levels observed. Acute bacterial rhinosinusitis patients receiving 20 mg EPs 7630 three times a day for ten days showed increased concentrations of MCP-1 and interferon gamma-induced protein 10 (IP-10) (*p* < 0.01) [65]. Furthermore, in post-viral rhinosinusitis patients receiving the same treatment, MCP-1, IP-10, and macrophage inflammatory protein (MIP)-1β were increased in nasal secretions [66]. The chemo- and cytokines MIP-1α, epithelial neutrophil activating peptide were increased both in acute and post-viral rhinosinusitis. This could indicate that EPs 7630 can regulate monocytes and neutrophils in the nasal mucosa and, hence, influence the infection [65,66].

##### Antimicrobial Proteins

Furthermore, EPs 7630 has been shown to increase the release of different antimicrobial proteins by different cells. In human epithelial cells, both infected with rhinovirus and uninfected, EPs 7630 has been shown to upregulate the expression of antimicrobial proteins such as β-defensin-1 and suppressor of cytokine signalling 1 [53]. EPs 7630 increased the release of the antibacterial proteins neutrophil peptides 1–3 (HNP 1–3) and bactericidal/permeability-increasing protein (BPI) from human neutrophils and granulocytes. After a five-hour incubation with EPs 7630 at a concentration of 30 µg/mL, HNP 1–3 was increased by 150% while BPI increased 127%. While 10 ng/mL LPS increased the release of HNPs 82% and BPI 356%, the combination of 30 µg/mL EPs 7630 and 10 ng/mL LPS stimulated the release even more and caused HNPs to increase by 294% and BPI by 531%. This could indicate that EPs 7630 can cause a stronger reaction against pathogens [62]. Furthermore, indirectly, by stimulating the release of IL-22, EPs 7630 can increase the production of antimicrobial protein S100A9 in lung tissue as explained before (see Section Cytokine Production and Effects on Other Immune Cells) [61].

#### 3.3.4. Expectorant Activity

Other effects of *P. sidoides* include effects on mucocilliary clearing. In mice that were intraperitoneally injected with phenol red, the elimination of the dye through the respiratory tract was dose-dependently increased with the administration of 20, 40, or 120 mg/kg bodyweight EPs 7630 for three days. This increase was statistically significant at doses of 40 and 120 mg/kg bodyweight when compared to mice receiving no treatment (*p* < 0.01) [54]. An underlying mechanism could be an increase in ciliary beat frequency of nasal epithelium cells. In an adherent monolayer cell culture of human nasal epithelium, EPs 7630 increased the frequency of ciliary beats by up to 123% at a concentration of 30 µg/mL and to 133% at a concentration of 100 µg/mL [57].

Antitussive effects were found in guinea pigs in a citric acid-induced cough model. A significant and dose-dependent reduction in the frequency of cough was observed in guinea pigs receiving 10, 20, or 45 mg/kg bodyweight EPs 7630 (*p* < 0.01). Moreover, in mice with ammonia-induced cough, a reduction in cough frequency as well as a prolongation of cough latency time was observed with doses of EPs 7630 of 20, 40, or 120 mg/kg bodyweight [54].

#### 3.3.5. Expert Opinion

*P. sidoides* is only used in preparations, the most well-known and well-researched being Kaloba^®^, and not on a more uncontrollable basis such as in a tea. This is an advantage, as these preparations are standardised and well researched. Clinical records are very good, and the mechanism is thought to be a combination of immune enhancement, antibacterial, antiviral, and expectorant effects. As most research is performed with the full extract, it is not possible to attribute the observed effects to one specific compound. There is an important role for coumarins, especially umckalin, but groups of compounds such as tannins and phenolics are also expected to contribute to the spasmolytic, antiphlogistic and antibacterial effects (Emeritus Prof. Dr. Liselotte Krenn, personal communication 2021).

A major issue with *P. sidoides* is, however, the lack of quality control. The European monographs use too broad a definition to ensure that actual *P. sidoides* is used. Related species such as *P. reniforme* and others can also be included in products sold as *P. sidoides*. The use of certain marker compounds, which is performed for example with standardised extracts such as EPs 7630, does not ensure that the same species is used either, as these marker compounds can still be present in other species. Overall, the chemical composition is likely different between different species, which is reflected by the fact that local people will not use any other species. Since almost all research is performed with EPs 7630, it is plausible, but not confirmed, that these effects would translate to other *P. sidoides* preparations (Mathias Schmidt, personal communication 2021).

### 3.4. Echinacea Species

Of Echinacea species different effects are known, including antibacterial, antiviral, immunomodulatory and expectorant effects. An overview of these is given in Table 3. Below they are discussed in further detail.

#### 3.4.1. Antibacterial Effects

Only one study has reported on the direct antibacterial effects of Echinacea. Here, it was found that a standardised alcoholic extract of *E. purpurea* (Echinaforce, A. Vogel Bioforce AG) reduced the expression of intracellular adhesion molecule 1 (ICAM-1) in human lung epithelial (BEAS-2B) cells. Subsequently, the adhesion of both *Staphylococcus aureus* and *Haemophilus influenzae* was reduced [70].

#### 3.4.2. Antiviral Effects

Some studies point to a direct antiviral effect of Echinacea [74]. For example, it was found that *E. purpurea* can directly inhibit influenza virus A (both human and avian), influenza virus B, herpes simplex virus 1 and 2, respiratory syncytial virus, and rhinoviruses [71]. Furthermore, in vitro pre-incubation of mouse fibroblasts with *E. purpurea* juice, methanolic extract, or aqueous extract for 24 h resulted in resistance to influenza A2, herpes, and vesicular stomatitis virus [72]. Another study found that the presentation of viral antigens by infected cells increased after exposure to *E. purpurea* juice in vitro. However, in this study, viral replication or viral load did not change [73]. When assessing light-activated antiviral activity, an n-hexane extract of *E. purpurea* had an MIC of 0.12 mg/mL, an ethanolic extract of E. pallida had an MIC of 26 µg/mL, and the isolated compound cichoric acid had an MIC of 45 µg/mL against HSV-1. E. angustifolia inhibits influenza A, herpes simplex virus 1, and rhinovirus, whilst *E. pallida* inhibits herpes simplex virus 1 and 2 [71].

The underlying mechanisms could involve inhibiting the influenza-virus-specific hemagglutinatinin and neuramidase [71]. At concentrations between 1.6 µg/mL and 1.6 mg/mL, a standardised ethanolic extract of *E. purpurea* (Echinaforce) inactivated human H1N1 type IV virus, highly pathogenic avian influenza virus (H5 and H7 types), and swine-origin influenza virus (S-OIV and H1N1) in cell culture assays. Direct contact between the virus and the extract was needed for this effect. Possibly this is because the extract inhibits the receptor binding activity of the virus, as was shown in hemagglutination assays. Interestingly, in sequential passage studies it was shown that in cell culture with H5N1 virus, no resistance of the virus to the *E. purpurea* extract developed, whilst this was the case with the control drug Tamiflu. Furthermore, the Tamiflu-resistant virus was still equally susceptible to the *E. purpurea* extract compared to the wild type virus [78]. Another underlying mechanism of the antiviral effect might be an increase in both the antibody-dependent and the innate NK-mediated activity, which has been found with *E. purpurea* extract and juice against herpes virus [73].

#### 3.4.3. Immunomodulatory Effects

The effects of Echinacea on the immune system are diverse. Depending on the circumstances, both immunostimulatory and anti-inflammatory effects have been observed.

##### Immunostimulatory Effects

Immunostimulatory effects have been reported on both non-specific and specific immunity. A stimulating effect was found in several immune cells. Firstly, *E. purpurea* can increase the total white blood cell count and specifically increase the proportions of lymphocytes and monocytes, whilst reducing neutrophil and eosinophil counts [72]. However, changes in T and B cell count are inconsistent [6,73]. Furthermore, *E. purpurea* can stimulate immune function by enhancing T-lymphocytes’ activity and neutrophils and macrophage-induced phagocytosis. It also possesses short-term, non-specific immune system stimulating properties such as increasing the activity of components of the alternate complement system [74,75,79]. These effects have also been confirmed in mechanistic clinical trials (see Section Evidence from Mechanistic Clinical Trials).

There are also reports of increased NK cell activity [72,74,76]. After 14 days of supplementation in mice, enhanced resistance of splenic lymphocytes to apoptosis was found [72]. Furthermore, a reduction in splenic NK cell activity as part of a stress response was prevented by Echinacea extract [76]. Moreover, in healthy people, patients with chronic fatigue syndrome, and AIDS patients an increase in NK cell activity was found after *E. purpurea* supplementation [77], although other studies did not find an increase in NK cells [72].

Other immunostimulant effects include, for example, both phenotypic and functional maturation of murine and human dendritic cells. It has also been found to reduce stress-induced reduction in splenocyte proliferation [76]. In addition, there is evidence that Echinacea can enhance macrophage functioning (in vitro, in vivo, and from human studies) as *E. purpurea*, E. pallida, and E. angustifolia all increased phagocytosis in vitro [77]. Furthermore, an increase in the levels of IL-1, IL-10, and TNF-α was reported (both in rat in vivo studies and in vitro macrophage studies) with the fresh juice of *E. purpurea* [72,73,74] which could be comparable to LPS simulation [77]. IL-6 and IFN-γ also increased [72,73,74,77]. This effect was especially observed with the polysaccharide fraction of *E. purpurea*. More specifically, an increase in the LPS-induced NO release by rat splenic macrophages was observed. Moreover, a dose-dependent increase in phagocytic activity of these macrophages was observed with an aqueous extract of an (unspecified) Echinacea extract [72]. In addition, stimulation of polymorphonuclear granulocytes was found with *E. purpurea* [73].

Furthermore, an immunostimulant effect is reflected in the increased levels of pro-inflammatory cytokines that has been reported in several studies. Echinacea species can, for example, increase IL-2 levels [72] as well as increase levels of TNF-α, IL-1α, IL-1β, IL-6, IFN-γ, and NO (in both rat in vivo studies and in vitro macrophage studies) [73,75]. It also can restore serum protein and splenic mRNA levels of cytokines in rats (IL-6, IL-10, and IL-17) and humans (IL-2, IL-16, IFN-γ, and TNF-a) [76]. With regard to different species, *E. purpurea* enhances IL-1, IL-6, TNF-α, and IFN-γ more than E. angustifolia or E. pallida [73].

However, the changes in cytokine levels also reflect the different effects Echinacea can have on the immune system. For example, an aqueous extract of *E. purpurea* (80% polysaccharides) enhanced dose-dependently the production of IL-2 and IFN-γ by human T-cells (Jurkat E6) when stimulated by phorbol 12-myristate 13-acetate plus ionomycin (PMA-I) in vitro. This stimulation was, however, absent when the cells were not stimulated by PMA-I. In conditions where the T-cells were seeded with a low density (0.5 × 106 cells/mL), *E. purpurea* also reduced the IFN-γ response and the percentage of CD25+ T-cells. This effect was, however, not seen when T-cells were seeded with a high density (5.0 × 106 cells/mL) [80]. Furthermore, in human lung epithelial cells (BEAS-2B), a standardised *E. purpurea* extract (Echinaforce) effectively prevented super-expression of inflammatory cytokines, otherwise known as a cytokine storm, in response to infection with influenza A virus. It achieved this by suppressing the expression of the NFκB pathway and possibly also TLR-4 [70].

##### Anti-Inflammatory Effects

More anti-inflammatory effects in epithelial tissues have been reported. For example, the expression of inflammatory cytokine genes and some of their properties in bronchial epithelial cells can be reversed by *E. purpurea*. Furthermore, undefined Echinacea extracts can inhibit the activity of COX-1 and COX-2 [72,73]. Cyclo-oxygenase (COX)-1, COX-2, and lipoxygenase enzymes are important enzymes involved in the production of pro-inflammatory prostaglandins. Inhibition of COX-1 and COX-2 was found with *E. purpurea*, whilst inhibition of 5-LOX was found with *E. purpurea*, E. pallida, and E. angustifolia. This inhibition of 5-LOX appears to be related to the concentration of alkamides [72]. Furthermore, lipoxygenase-inhibiting anti-inflammatory activity was attributable to isobutyl amide and decatetraene acid in *E. purpurea* [76]. In addition, inhibition of hyaluronidase is reported as an anti-inflammatory effect of Echinacea species, caused by cichoric acid as the most potent inhibitor [73].

##### Evidence from Mechanistic Clinical Trials

The effect of Echinacea species on the immune system is also observed in mechanistic clinical trials that found changes in white blood cell count, cytokine levels, and the complement system. For example, a seven day treatment with 5 mL of a standardized *E. purpurea* extract (Echinilin) increased the total white blood cell, monocyte, neutrophil, and NK cell counts significantly in healthy volunteers [81].

Changes in cytokine levels were also observed with daily supplementation with an *E. angustifolia* syrup (corresponding to 4.7 mg Echinacoside and 8.0 mg of a high-molecular-weight (20 kDa) polysaccharide). This caused an increase in IL-2 and IL-8 mRNA levels and a decrease in TNF-α and IL-6 mRNA levels in healthy volunteers (*n* = 10). The changes in IL-2 and IL-6 were confirmed by protein levels of these cytokines in the blood. Supplementation went on for a month, but these effects were maximal after 14 days [82].

In another study, healthy volunteers (*n* = 30) received supplementation with a standardised ethanolic *E. purpurea* extract (Echinaforce) for eight days. Afterwards, blood samples were taken and stimulated in vitro with LPS, SEB, or zymosan. The pro-inflammatory mediators TNF-α and IL-1β decreased by up to 24% (*p* < 0.05) while anti-inflammatory IL-10 levels increased by 13% (*p* < 0.05), and IL-8 and MCP-1 increased by 15% (*p* < 0.05) when compared to baseline measurements. In a subgroup of volunteers who showed low levels of MCP-1, IL-8, IL-10, and IFN-γ, treatment with Echinaforce significantly increased these levels (30–49%, *p* < 0.05), whereas volunteers with higher levels of these cytokines before treatment did not show any significant change in their levels after treatment. Furthermore, in volunteers with reported high stress levels and who experienced more than two colds per year, treatment increased IFN-γ levels (>50%). A relationship with stress was also found in volunteers with a low level of cortisol, in whom levels of IL-1β, IL-6, IL-12, and TNF-α decreased with treatment, while in volunteers with higher cortisol levels such changes were not found [83].

Furthermore, after four weeks of taking daily a combination of *E. purpurea* and *E. angustifolia* (*E. purpurea* whole herb extract 4% (908 mg/day), *E. purpurea* whole herb (464 mg/day) and *E. angustifolia* root (36 mg/day)), complement properdin increased by 21% (*p* < 0.05) in healthy volunteers (*n* = 8) compared to a placebo treatment [84].

##### Compounds Involved and Differences between Species

Some studies have also looked specifically at compounds that could be involved in the immune modulating effects. Many point to the polysaccharides as important for this. Intravenous injection with the polysaccharide fraction was, for example, twice as effective compared to the whole aqueous extract in reducing carrageenan-induced rat paw oedema. It was half as effective as indomectin in the croton oil test when applied topically [72]. Polysaccharides have further been shown to enhance phagocyte function in in vitro and in vivo studies by increasing phagocytosis, increasing TNF-α, IL-1, and IL-6 secretion. Furthermore, they increased the motility of polymorphonuclear cells and their cytotoxic activity [77]. Finally, the enhanced immune response of human T-cells to PMA-I was also found with the polysaccharides and not with phenolic compounds in *E. purpurea* [80].

Moreover, alkamides derived from Echinacea have immunomodulatory and anti-inflammatory activity. This is reflected in the inhibiting effect of alcohol extracts of Echinacea on the production of inflammatory mediators such as TNF-α and NO [75]. The alkylamides dodeca-2E,4E,8Z,10E/Z-tetraenoic acid isobutylamides, trienoic, and dienoic acid derivatives in *E. purpurea* increased TNF-α mRNA synthesis in primary human monocytes and macrophages through activation of the endocannabinoid receptor 2, though no increase in TNF-α protein levels was found [85].

With regards to the different species of Echinacea, the evidence differs. For example, arabinogalactan-proteins from *E. pallida* strongly stimulated proliferation of mouse lymphocytes in vitro, while the arabinogalactans from *E. purpurea* did not show this effect [86]. Furthermore, cichoric acid, which is involved in immunomodulation, enhancing phagocytosis, and inhibiting hyaluronidase, is the main phenolic compound in *E. purpurea*. Echinacoside is the main phenolic compound in *E. pallida* and *E. angustifolia* and does not have immunostimulatory effects, though anti-inflammatory and antioxidant properties have been reported [87].

#### 3.4.4. Expectorant Effects

Aside from a direct virucidal effect and an immunomodulatory effect, *E. purpurea* reduced excessive mucin production by airway cells and tissues [71].

#### 3.4.5. Expert Opinion

Regarding Echinacea, the main effect in mechanistic studies appears to be immunomodulation. There are some conflicting results, but this could be due to a U-shaped response curve, where in low concentrations the effects are more immunomodulatory, whilst in higher concentrations the effect is more immunosuppressive. The reported changes in white blood cell count are inconsistent. More so, animal studies are susceptible for finding such changes, often inconsistent, and the translation to clinically relevant effects is not clear. Increased functioning of macrophages is a mechanism that can enhance immune function but is difficult to prove in clinical studies (Emeritus Prof. Dr. Liselotte Krenn, personal communication 2021) and in vitro and in vivo models do not represent the situation of infection very well (Dr. Mathias Schmidt, personal communication 2021). Furthermore, contaminations with endotoxins can easily occur with testing Echinacea in vitro. These can have an LPS-like activity and can contaminate the results, leading to an unjustified conclusion of immunostimulation (Prof. Dr. Roman Huber, personal communication 2021).

### 3.5. Hedera helix L.

Of *H. helix* different effects are known, including antibacterial, antiviral, immunomodulatory, bronchospasmolytic and secretolytic effects. An overview of these is given in Table 4. Below they are discussed in further detail.

#### 3.5.1. Antibacterial and Antiviral Effects

A direct antibacterial effect of an aqueous extract of *H. helix* leaves was assessed for *S. pneumonia*, *S. pyogenes*, *S. aureus*, *S. epidermidis*, *M. tuberculosis*, *M. avium*, *H. influenza*, and *A. baumannii*. The MICs ranged from 16 to 64 μg/mL, with the lowest MICs (16 μg/mL) found with *M. avium* and *H. influenzae* [88]. The petroleum and ethanolic extracts were found to be less effective against *S. aureus*, *S. epidermidis*, *E. coli*, *K. pneumoniae*, *P. auruginosa*, and *P. mirabilis*, with MICs of 156.2 μg/mL or higher [89]. No reports on direct antiviral effects were found.

#### 3.5.2. Anti-Inflammatory Effects

Treatment with *H. helix* extracts has been shown to reduce the level of pro-inflammatory cytokines. In murine macrophages (J774.2 cells), significant decreases in IL-6 were observed of 25 ± 4%, 32 ± 4%, and 40 ± 7% with concentrations of 80, 160, and 400 μg/mL EA575, respectively [90]. In both J774.2 and HEK293 cells, the anti-inflammatory effects have been shown to be a result of a dose-dependent inhibition of the NFkB pathway, probably due to a specificity switch of IKKβ [91]. In in vivo studies, an anti-inflammatory effect was also observed for saponins present in *H. helix*. In ovalbumin-sensitized guinea pigs, for example, a peritoneal injection of either a high (3 mg/kg bodyweight) or a low dose (0.3 mg/kg bodyweight) of α-hederin reduced total white blood cell and eosinophil counts compared to sensitized animals without treatment (*p* < 0.01 and *p* < 0.001, respectively). There was an increase in the percentage of neutrophils, lymphocytes, and monocytes, whilst the eosinophil and basophil counts decreased (*p* < 0.01–0.05) [92]. Moreover, blood levels of IL-4 and IL-17 were decreased with the high dose (*p* < 0.05), while INF-γ levels increased with both low and high doses compared to the control (*p* < 0.05) [93].

Furthermore, treatment with α-hederin reduced pathological changes in lung tissue due to asthma induction, such as reduced thickness of vessels and airway wall, mucus plaque, and respiratory epithelium denudation [93]. Similarly, in ovalbumin-sensitized BALB/c mice, treatment with *H. helix* (100 mg/kg) reduced the increased number of goblet cells and reduced the increased thickness of basement membrane. However, compared to dexamethasone only the reduction in thickness of the basement membrane was greater with *H. helix* while other histopathological parameters improved more with dexamethasone [94].

Looking at different compounds that could be involved in an anti-inflammatory effect, oral administration of 0.02 mg/kg α-hederin was found to be ineffective for reducing carrageenan-induced rat paw oedema after one hour. The same was found for hederasaponin-C, while hederacolchisides-E and -F showed slight anti-inflammatory effects after one hour (*p* < 0.05). After four hours, hederacolchiside-F was found as effective as the control drug indomethacin (20 mg/kg), while hederacolchisides-C and -E were both effective, though less effective compared to indomethacin [99].

#### 3.5.3. Bronchospasmolytic and Secretolytic Effects

Some studies have found that *H. helix* can relax the smooth muscle in the lungs, leading to bronchodilation. In vitro, the three saponins α-hederin, hederagenin, and hederacoside C did not influence histamine- or methacholine-induced contraction in muscle strips. Pretreatment with 1 µM α-hederin for 18 h, however, enhanced isoprenaline-induced relaxation. Hederagenin and hederacoside C did not show this effect [95]. In vivo, these findings were confirmed. In ovalbumin-sensitized guinea pigs, intraperitoneal injection with either a high (3 mg/kg bodyweight) or low dose (0.3 mg/kg bodyweight) of α-hederin decreased the responsiveness of tracheal smooth muscle to methacholine, histamine, and ovalbumine. The change in responsiveness to ovalbumin was even stronger than with the control treatment thymoquinone [92].

An underlying mechanism causing these effects appears to be that *H. helix* extract can reduce the internalization of β2 adrenergic receptors. This was shown with pre-incubation of human embryonic kidney (HEK293) cells with 1 µM α-hederin or 1 µM β-hederin for 24 h. In addition, in type 2 alveolar cells (A549 cells) pre-treated with 1 µM α-hederin or 1 µM β-hederin, binding of a dye (Alexa532-NA) to the β2 adrenergic receptor increased. Interestingly, in saturation experiments, α-hederin did not change the density of β2 adrenergic receptors. Furthermore, in human airway smooth muscle (HASM) cells, pretreatment with 1 µM α-hederin or 1 µM β-hederin for 24 h increased cAMP levels when the cells were stimulated compared to no pretreatment [96,97]. An increase in cAMP can indicate (increased) activation of the β2 adrenergic receptor [98].

These results indicate that pretreatment with α-hederin prevents the number of β2 adrenergic receptors from declining under stimulating conditions. This increased responsiveness could lead to a bronchospasmolytic effect in the smooth muscle cells. In alveolar cells, this increased stimulation of the β2 adrenergic receptor can lead to increased secretion of surfactant. This can cause the bronchial mucus to be diluted and more easily expelled [98]. 

The effect on β2 adrenergic receptors is only found with α-hederin and β-hederin. Hederacosid-C and hederagenin did not show these effects on β2 adrenergic receptor internalization [97]. Protocatechuic acid, neochlorogenic acid, chlorogenic acid, cryptochlorogenic acid, rutin, kaempferol-3-O-rutinoside, 3,4-, 3,5-, and 4,5-dicaffeoylquinic acid, and hederacoside B did not affect β2 adrenergic receptors [96]. However, resorption studies have shown that hederacoside C does not reach significant levels in the blood but is readily transformed into α-hederin instead. It can, therefore, be seen as a prodrug. Furthermore, human resorption studies have shown that α-hederin reaches blood plasma concentrations of 0.66 µM, whilst concentrations of 0.5 µM have been shown to induce an inhibition of approximately 60% of the β2 adrenergic receptor internalization [98].

### 3.6. Primula veris L. and Primula elatior L.

No reports on the effects of isolated *P. veris* or *P. elatior* were found.

### 3.7. Thymus vulgaris L. and Thymus zygis L.

#### 3.7.1. Antibacterial Effects

In vitro studies assessing the antibacterial effects have focused on different extracts and some on individual compounds. Antibacterial effects have been shown against a broad spectrum of bacteria, including those relevant to URTI. In the following paragraphs, ethanolic extracts, essential oil, and individual compounds, will be discussed.

##### Ethanolic Extracts

First, there are studies into the ethanolic extract of *T. vulgaris*. Using disk diffusion, MICs against *S. aureus* of 5 mg/mL, against *E. coli* and *K. pneumoniae* of 10 mg/mL, and against *P. vulgaris* and *P. auruginosa* of 20 mg/mL were established [100]. Furthermore, a 25 mg/mL ethanolic extract showed a zone of inhibition ranging from 4.00 to 21.00 mm against ten multidrug-resistant clinical isolates of *S. aureus*, *E. coli*, *P. mirabellis*, and *K. pneumoniae* and two standard strains: *S. aureus* ATCC 25923 and *E. coli* ATCC 25922 [101].

##### Essential Oils

Most research on the direct antibacterial effects of *T. vulgaris* and *T. zygis* has, however, focused on the essential oils. The antibacterial effects of the essential oil has, for example, been shown against *P. fluorescens*, *L. monocytogenes*, *B. thermosphacta*, *E. coli*, *Salmonella*, *P. auruginosa*, and *S. aureus* [102,103,104,105]. Overall, Gram-positive bacteria appear to be more vulnerable compared to Gram-negative [106]. An overview of the different MICs that have been established with different methods is given in Table 5. Toxicity against non-bacterial cells was tested and a minimum non-toxic concentration to Vero cells was found of 0.0002 mL/mL [107].

Some studies also investigated the antibacterial effect of the essential oil of *T. vulgaris* against some standard-drug-resistant bacteria. Though it showed activity against some multidrug-resistant bacteria such as MRSA, to others, such as resistant *P. aeruginosa*, it did not [108]. Interaction with standard antibiotics was also investigated against extended-spectrum β-lactamase (ESBL)-producing and New Delhi metallo-β-lactamase (MBL)-1-producing *K. pneumoniae* isolates. Here, the combination of the essential oil of *T. vulgaris* with gentamicin had an additive effect rather than a synergistic effect [109].

Furthermore, the essential oil of *T. zygis* was tested against several bacterial strains as well. MIC values against a collection of colistin-sensitive *A. baumanii* and *K. pneumoniae* ranged between 256 and 1024 µg/mL. Synergy was observed against these organisms when the essential oil was given in combination with colistin, but not with other antibiotics [110].

**Table 5:** Overview of the minimum inhibitory concentration (MIC) values that have been established with direct contact for essential oil of *T. vulgaris*.

Bacterial Species	Method	MIC	
*Acinetobacter baumanii*	Agar dilution	0.12% (*v*/*v*)	[111]
*Enterococcus faecalis*	Agar dilution	0.5% (*v*/*v*)	[111]
*Aeromonas sobria*	Agar dilution	0.12% (*v*/*v*)	[111]
*Escherichia coli*	Agar dilution	0.12% (*v*/*v*)	[111]
	Broth microdilution	0.03% (*v*/*v*)	[111]
	Microwell dilution	62.5 µg/mL	[112]
*Klebsiella pneumoniae*	Agar dilution	0.25% (*v*/*v*)	[111]
	Microwell dilution	500 µg/mL	[112]
		0.025 mL/mL	[107]
*Pseudomonas aeruginosa*	Agar dilution	>2.0% (*v*/*v*)	[111]
	Microwell dilution	>500 µg/mL	[112]
*Salmonella typhimurium*	Agar dilution	>2.0% (*v*/*v*)	[111]
	Microwell dilution	125 µg/mL	[112]
*Serratia marcescens*	Agar dilution	0.25% (*v*/*v*)	[111]
*Staphylococcus aureus*	Agar dilution	0.25% (*v*/*v*)	[111]
	Broth microdilution	0.03% (*v*/*v*)	[111]
		0.0125 mL/mL	[107]
	Microwell dilution	31.2 µg/mL	[112]
*Streptococcus pyogenes*	Broth microdilution	0.43 mg/mL	[113]
		0.0125 mL/mL	[107]
*Streptococcus pneumoniae*	Broth microdilution	0.11 mg/mL	[113]
		0.00625 mL/mL	[107]
*Streptococcus mutans*	Broth microdilution	0.04 mg/mL	[113]
*Haemophilius influenzae*	Broth microdilution	0.11 mg/mL	[113]
		0.00625 mL/mL	[107]
*Haemophilius parainfluenzae*	Broth microdilution	0.11 mg/mL	[113]
*Moraxella catarrhalis*	Broth microdilution	0.09 mg/mL	[113]
*Meticillin-resistant Staphylococcus aureus* (*MRSA*)	Tube dilution	0.4 mg/mL	[108]
*Pseudomonas aeruginosa*	Tube dilution	1.4 mg/mL	[108]
*Bacillus cereus*	Microwell dilution	15.6 µg/mL	[112]
*Proteus vulgaris*	Microwell dilution	31.2 µg/mL	[112]
*Salmonella typhi*	Microwell dilution	250 µg/mL	[112]
*Streptococcus agalactiae*	Broth microdilution	0.00625 mL/mL	[107]
*Stenotrophomonas maltophilia*	Broth microdilution	0.003125 mL/mL	[107]
Extended-spectrum beta-lactamase (ESBL)-producing *K. pneumoniae*	Broth microdilution	3.6 mg/mL	[109]
New Delhi metallo-beta-lactamase (MBL)-1-producing *K. pneumoniae*	Broth microdilution	5.4 mg/mL	[109]

##### Compounds

The essential oil of *T. vulgaris* consists of several compounds that could have antibiotic effects. An analysis showed that the essential oil contained about 43.3% thymol, 25.5% p-cymene, and 4.1% carvacrol [114]. Thymol in particular has been researched extensively, and possesses antibacterial activity against a large range of species, including biofilm-embedded ones [32,115]. P-cymene did not show much antibacterial activity against Gram-negative bacteria, whereas carvacrol appeared to be effective against *S. aureus*, *E. faecalis*, *E. coli*, *P. aerigunosa*, *P. vulgaris*, *K. pneumoniae*, and *Salmonella* sp. [114]. Thymol, eugenol, and cavacrol have also been shown to inhibit food borne pathogens [102].

#### 3.7.2. Antiviral Effects

Only a few studies have reported the antiviral effects of *T. vulgaris* or *T. zygis*. Ethanolic extract of thyme (species not specified) was found to reduce the cytopathic effect caused by influenza A virus dose-dependently at non-toxic concentrations (0.03–0.33% *v*/*v*) in MDCK cells. Against rhinoviruses, no effect was observed [50]. Furthermore, a *T. vulgaris* aqueous extract was effective at inhibiting avian infectious bronchitis virus infection in Vero cells, with an EC50 value of 63.1 µg [116]. Furthermore, the essential oil of *T. vulgaris* has shown intercellular antiviral effects against influenza type A virus (H1N1) [102].

#### 3.7.3. Immunomodulatory Effects

Only a few articles have reported the effects on the immune system. In an acute pneumonitis mouse model, inhalation of the essential oil of *T. vulgaris* (maximum concentration 6.55 µL/L) reduced inflammatory airway hyperresponsiveness and myeloperoxidase activity. This was also reflected in a reduction in histopathological changes in the lungs [117]. Furthermore, thymol has also been shown to have anti-inflammatory effects in carrageenan-induced paw oedema in rats. A dose of 200 mg/kg bodyweight of thymol reduced the oedema equally to 300 mg/kg bodyweight acetylsalicylic acid. Leukocyte migration into the peritoneal cavity was also reduced with doses of 50 mg/kg bodyweight and higher [32].

### 3.8. Combinations of Ivy, Primrose, and Thyme

Looking at studies that investigated combinations of ivy, primrose, and thyme, one study looked into the effects of film-coated tablets with a combination of thyme and primrose in a ratio of 2.67:1 (Bronchipret). It involved rats in which pulmonary inflammation was induced by LPS. When they received either 14, 68, or 339 mg/kg bodyweight per day (corresponding to 0.2, 1, and 5 times the human daily dose) orally of this combination, the infiltration of the lungs by inflammatory cells, myeloperoxidase (MPO) activity, and mucin 5A (MUC5AC) protein secretion in the lungs was significantly reduced after 48 h (*p* < 0.05). When using higher doses (68, 203, and 678 mg/kg bodyweight per day), not only MPO activity and MUC5AC secretion was reduced, but also goblet cell hyperplasia was partially prevented after 72 h. The reduction in MUC5AC secretion was confirmed in an in vitro model using human respiratory epithelium and Calu-3 cells, which were exposed to either 1 or 10 µg/mL for 14 days of this combination of thyme and primrose. Here, also mRNA levels of MUC5AC were reduced [118].

The same combination of thyme and primrose (Bronchipret, Bionorica SE, Neumarkt, Germany) was shown to inhibit arachidonate 5-lipoxygenase (LO-5) in an enzymatic assay. This inhibits the biosynthesis of leukotriene, which was confirmed in an in vivo study with rats receiving the same doses as described above. This animal study also confirmed the reduction in leukocyte infiltration of the lungs, as was found similarly in the previously discussed study [119].

Furthermore, a syrup consisting of thyme and *H. helix* in a ratio of 10:1 (Bronchipret) reversed the increase in leukocyte and goblet cell numbers in lung tissue in rats with LPS-induced broncho alveolitis. The syrup was given 1, 25, and 49 h after induction and the effects were observed with all three doses: 1.7, 5.0, and 16.7 mL/kg bodyweight. The syrup also prevented an increase in bronchoalveolar lavage fluid and blood. Furthermore, in vitro this syrup reduced the levels of leukotriene B4, cysteine leukotriene receptor, and 5-lipoxygenase with IC50 values of 36 µg/mL, 10 µg/mL, and 19 µg/mL, respectively [120].

#### Expert Opinion

Thyme contains essential oils that are antibacterial and could thus aid in respiratory tract infections. Other compounds in thyme, such as flavonoids and tannins, could also contribute to an antibacterial effect. Especially tannins of the rosmarinic acid type could act as spasmolytics. Primrose is very little researched, and its use is mostly based on traditional use. However, the saponins in primrose are very similar in structure to those in ivy and, therefore, it can be expected that they too can have a secretolytic effect. Both can slightly irritate the mucosa and, hence, stimulate it to dilute the mucus (Emeritus Prof. Dr. Liselotte Krenn, personal communication 2021). Saponins in primrose, furthermore, irritate the vagus nerve in the stomach. This results in a response in the entire body, including the respiratory tract, where it acts as a secretolytic. Hence, it dilutes the mucus and eases expectoration. Thyme acts as an antimicrobial in in vitro studies. However, it is not yet clear how thyme constituents end up in the respiratory tract when it is taken orally (Dr. Mathias Schmidt, personal communication 2021). Combinations with ivy, primrose, and thyme create a useful mixture that has antimicrobial and secretolytic effects (Prof. Dr. Roman Huber and Dr. Mathias Schmidt, personal communication 2021).

## 4. Discussion

TCIH treatments offer a unique perspective and potential opportunities for reducing the inappropriate use of antibiotics, and so to address antimicrobial resistance. This systematic literature review focusses on the underlying mechanisms of four TCIH treatments that have shown moderate- to high-quality evidence of clinical effectiveness in the treatment of URTIs: *Andrographis paniculata*, *Pelargonium sidoides*, *Echinacea* species, and the combination of ivy, primrose, and thyme [16,17,18,19,20,21,22,23]. The mechanisms were hypothesized to include not only direct antibacterial and antiviral effects, but also the immune response of the patient and the modulation of URTI symptoms such as cough and fever [13].

### 4.1. Andrographis paniculata

For *Andrographis paniculata*, the beneficial effects appear to result from a combination of antibacterial, antiviral, anti-inflammatory, and possibly antipyretic activity. Antiviral effects have been shown both in vitro and in vivo with clinically realistic doses [38,39] and are hence a likely underlying mechanism. Furthermore, there is an interesting indication from animal studies that andrographolide can reduce inflammation (measured by IL-6 and TNF-α), whilst keeping sufficient inflammatory activity to fight off an infection [35]. The clinical relevance of direct antimicrobial effects is as yet hard to assess. It was mostly investigated by agar well diffusion assays and these give only limited insight into the size of the effect [24,33]. The MICs that have been reported are all above 100 µg/mL [35] which, according to expert opinion, suggests that these effects are not clinically relevant. On the other hand, indirect antibacterial effects such as sensitisation to antibiotics and reduction in adherence to host epithelium could indicate an overall antibacterial effect [35]. Finally, antipyretic effects are shown in only one study and this study used high doses. As it is still unknown by what molecular mechanism this inhibition is caused, it is not yet possible to assess if and in which cases of URTI this effect is beneficial [13]. More research into this mechanism is, therefore, recommended [24].

### 4.2. Pelargonium sidoides

There is interesting evidence that the standardised ethanolic extract from *P. sidoides*, EPs 7630, increases the adherence of bacteria to immune cells and simultaneously decreases the adherence to host epithelial cells [48,49]. In terms of immunomodulation *P. sidoides* reduces infection-related tissue damage during infection (e.g., inflammatory damage to the lungs), hence can reduce sickness behaviour [51,52,54,55,56]. On the other hand, it increases antimicrobial functioning of macrophages in vitro [48,59,60,61,63,64,66,69]. Furthermore, *P. sidoides* also has a direct antiviral effect, possibly involving upregulation of the vitamin D receptor [50,51,52], and expectorant effects [54,57], and thus contributes to URTI treatment. Based on expert opinion, direct antibacterial effects are not considered as clinically relevant as MICs are above 100 µg/mL. However, the stimulation of the immune system to produce antimicrobial proteins could be an indirect antibacterial effect in practice [52,61,62]. This and the antiviral effects could reduce the duration of URTI symptoms, while the reduction in inflammatory damage and expectorant effects could help to resolve URTI symptoms such as sore throat and cough.

### 4.3. Echinacea Species

Both the literature and the experts agree that the effect of *E. purpurea* is mostly immunomodulatory. However, none of the experts was fully convinced of its efficacy, and interpreting the different findings remains difficult since both anti-inflammatory and immunostimulatory effects have been reported in the literature. Immunostimulatory measurements are hard to model in vivo. In vitro assays are vulnerable to endotoxin contamination, and are not necessarily directly translatable to a live situation. From mechanistic clinical trials, the effect in healthy volunteers appears to be mostly anti-inflammatory, though differences in immune response between healthy and immunosuppressed people indicate that the effect can change in a different immunological situation [81,82,83,84]. Different effects in people with different immunological states might explain the inconclusive and sometimes contradictory findings in clinical trials. Moreover, antiviral effects are plausible as extracellular antiviral effects against inactivated human H1N1 type IV virus start at concentrations of 1.6 µg/mL ethanolic *E. purpurea* extract [71,72,73,74,75]. This is especially interesting as in cell culture assays resistance did not occur, whilst it did occur with the standard drug Tamiflu [78]. There is no good evidence to suggest a clinically relevant antibacterial effect. For the other species, *E. pallida* and *E. angustifolia*, similar effects are expected, though not enough studies were retrieved to confirm this. Furthermore, the variety in species and preparation methods used means it is not possible to draw strong conclusions as it is possible that the variability in the results is caused by differences between preparations.

### 4.4. Ivy, Primrose, and Thyme

The effect of ivy, primrose, and thyme in the treatment of cough is explained by an additive effect of their individual effects. First, ivy can prevent internalization of β-adrenergic receptors, and thus causes a secretolytic effect [92,95,96,97,98]. The resulting bronchodilation can decrease the airway resistance and, hence, improve airflow in the lungs and reduce shortness of breath. The increased amount of surfactant dilutes the mucus and eases its expulsion. According to expert opinion, saponins in primrose might act in a similar way or might stimulate the vagus nerve to bring about a secretolytic effect, but we found no evidence in the literature for this. The added value of primrose in this mixture, and whether potential side effects would outweigh the positive effects or not, therefore, remains unsure. Finally, *T. vulgaris* acts mainly via direct antibacterial effects of the essential oil [102,106,107,108,109,110,121]. According to expert opinion, these effects are promising as many reported MIC values are under 100 µg/mL. There are no reported interactions between ivy, primrose, and thyme. Hence, there is no evidence that using them in a mixture should be preferred over using the three herbs separately. However, when combined each of them may contribute to a multi-targeted treatment that has bronchospasmolytic, secretolytic, and antibacterial effects.

### 4.5. Practical Implications

These findings give an overview of the mechanistic evidence and (possible) modes of action of effects that have been observed in clinical trials [13]. This creates a basis for clinical advice on the use of the investigated herbs in URTIs. However, before strong recommendations can be made, better defined descriptions of the treatments and their quality control are needed. Especially with *Echinacea*, there is a wide variety of products using different species of *Echinacea*, different plant parts, different extraction mediums and different extraction methods. As this results in many different products with different compositions, findings about one of these products cannot automatically be generalised to all. On the other hand, in the case of *P. sidoides* and *H. helix*, most research is performed using the standardised extracts EPs 7630 and EA 575. Consecutive studies with these extracts can be compared, which allows for a recommendation on their clinical use.

All four herbs hold great potential to aid in the reduction in the use of antibiotics. In the first place, they offer an alternative treatment option for URTI treatment [2]. Ways to ensure that new antibiotic treatments are less susceptible for developing AMR include using multi-target treatments, treatments that decrease virulence, or treatments that improve innate immunity [122]. Both *A. paniculata* and *P. sidoides* by themselves target different mechanisms, and the combination of thyme with either ivy or primrose is expected to be a multi-target treatment as well, targeting both the β-adrenergic receptor and directly targeting the bacteria. Secondly, compounds in *A. paniculata* and *T. vulgaris* may improve the effectiveness of standard antibiotics and, thus, might reduce the use of antibiotics [2]. Thirdly, *A. paniculata*, *P. sidoides* and *Echinacea* species may all reduce infection-related tissue damage, while both *P. sidoides* and *Echinacea* species are also suggested to increase the innate immune response. Therefore, all of these are promising new treatments for URTIs, especially *P. sidoides*, as it appears to be multi-targeted, decreasing infection-related tissue damage and modulating innate immunity.

### 4.6. Strengths and Limitations

While many studies on AMR only focus on targeting pathogens, this study also considered other possible mechanisms by which URTIs could be tackled, such as immunomodulation. Another strength of this study is that it was conducted systematically, with a transparent methodology and used many databases. Furthermore, study selection was performed independently by two authors, after which the findings were compared. Finally, findings from the literature were compared with and supplemented by interviews with different experts in this field.

However, some limitations of this study should be taken into consideration as well. The first is that, due to the varied methodology of studies of interest for the research question, setting uniform quality criteria was challenging. Due to time limitations and to avoid excessive exclusion general criteria were not established and applied in this study. Stronger conclusions might be drawn if quality standards of the tested products were introduced as inclusion criteria. Secondly, the search terms were based on several proposed mechanisms. It could be, however, that additional mechanistic pathways are involved, but were not retrieved as they were not included in the initial search terms. Furthermore, studies that were not written in English, German, or Dutch were excluded which might have left out relevant information published in articles in other languages. Finally, none of the interviewed experts had experience with *A. paniculata*. A larger panel of experts, with a broader experience, might have improved this aspect of the project.

## 5. Conclusions

*A. paniculata* probably acts mainly through immunomodulation and antiviral activity, and possibly also through antibacterial and antipyretic effects. *P. sidoides* most likely acts through antiviral, indirect antibacterial, anti-virulence, and expectorant effects, and *Echinacea* species most likely act through immunomodulation. The combination of ivy, primrose, and thyme combines secretolytic, spasmolytic, and antibacterial effects. This mechanistic evidence supports the promising data from clinical trials and opens the door to evidence-based recommendations for the use of these herbs in clinical URTI treatment. Furthermore, it offers suggestions for future research on the working mechanisms of other potential URTI TCIH treatments.

## 6. Future Directions

For the clinical use of the researched herbs, strong(er) quality standards should be developed to streamline research and allow for evidence-based recommendations. Published data on the effects of extracts need to be carefully checked for an authentic botanical source, the plant parts processed, and the specific production procedure.

Furthermore, since this study focussed on only two of the three pillars for evidence based medicine, (1) research evidence, (2) expert opinion, and (3) patient values [123], future research should pay more attention to the third pillar, ‘patient values’. Clinical trials seem to show that the treatments are effective [13]. However, the experience of patients with the treatments, the side effects, and the costs should also be taken into account.

Since there are indications that certain effects only occur at higher doses, more research into the dose–response relationship with all treatments is needed. Furthermore, toxicity, pharmacodynamics, and potential interactions with medications or other herbs should be monitored to prevent harmful side effects, and be taken into account when making recommendations for clinical practice. Further details on the mechanisms should also be researched, such as specific working mechanisms of individual active compounds. Pharmacodynamics is especially relevant as this type of research could determine if in the in vitro studies compounds are used that actually are absorbed and end up at the researched site.

Finally, the fact that the identified mechanisms occur in the studied treatments, suggests that these mechanisms should also be researched in other TCIH URTI treatments. These include treatments involving alternative antibacterial mechanisms that target the bacteria–host interaction, such as changes in adherence or the production of antimicrobial proteins. Given the lack of evidence retrieved on the working mechanisms of *P. veris* and *P. elatior*, this kind of research is recommended for these herbs in the future.

## Figures and Tables

**Table 1:** Overview of working mechanisms of *Andrographis paniculate* (*Burm. F*) *Nees*.

Antibacterial	Antiviral	Immunomodulatory	Other
Direct effects against *S. aureus*, *E. coli*, *S. typhimurium*, *B. subtilis*, *E. faecalis*, *K. pneumoniae*, *S. pneumoniae*, *P. vulgaris* [24,33,34,35,36,37]	Against avian influenza A virus (H9N2, H5N1 and H1N1) [38,39]	Increased proliferation of white blood cells [40]	Antipyretic [24]
Increased antibiotic sensitivity and reduced biofilm formation [35]		Reduced inflammatory response macrophages [41,42,43,44]	
Reduced bacterial adhesion to epithelium [35]		Anti-inflammatory [45,46]	

**Table 2:** Overview of working mechanisms of *Pelargonium Sidoides* (*Thunb.*) *R. Knuth*.

Antibacterial	Antiviral	Immunomodulatory	Other
Reduced bacterial adhesion to epithelial cells [48,49]	Against influenza A virus (H1N1 and H3N2), respiratory syncytial virus (HCo-229E), parainfluenza virus type 3 and coxsackievirus A9 [50,51,52,53]	Reduced inflammatory damage [50,53,54,55,56]	Expectorant [54,57]
Increased bacterial adhesion to buccal cells [48]	Downregulation of docking proteins infected bronchial epithelial cells [51,52]	Increased macrophage functioning [58,59,60]	Antitussive [54]
Increased production of antimicrobial peptides [53,61,62]	Prevention of hemagglutination in human erythrocytes [50]	Increased inflammatory cytokines [60,61,63,64,65,66]	
	Upregulation of vitamin D receptor on human epithelial cells [53]	Increased production of antimicrobial peptides [53,61,62]	

**Table 3:** Overview of mechanisms of *Echinacea* species.

Antibacterial	Antiviral	Immunomodulatory	Other
Reduced adhesion of *S. aureus* and *H. influenzae* to epithelial cells [70]	Against influenza virus A (both human and avian), influenza virus B, herpes simplex virus 1 and 2, respiratory syncytial virus and rhinoviruses [71]	Immunostimulatory effects: ○ Increased total white blood cell count [72,73] ○ Increased activity of T-lymphocytes, NK-cells, dendritic cells and macrophages [72,74,75,76,77] ○ Increased secretion of pro-inflammatory cytokines [72,73,74,75,76,77]	Expectorant [71]
	Increased presentation of viral antigens by infected cells [73]	Anti-inflammatory effects:Inhibition of COX-1, COX-2 and 5-LOX [72,73,76]	
	Decreased viral binding [71,78]		
	Increased antibody-dependent and innate NK-mediated activity [73]		

**Table 4:** Overview of mechanisms of *Hedera helix* L.

Antibacterial	Antiviral	Immunomodulatory	Other
Direct effects against *S. pneumonia*, *S. pyogenes*, *S. aureus*, *S. epidermidis*, *M. tuberculosis*, *M. avium*, *H. influenzae*, and *A. baumannii* [88,89]	No antiviral effects reported.	Anti-inflammatory effects [90,91,92,93] Reduced pathological changes to lung tissue [93,94]	Bronchospasmolytic and secretolytic [95,96,97,98]

## Data Availability

The data presented in this study are available in the Supplementary Files Table S1: Study selection.

## Supplementary Materials

The following supporting information can be downloaded at: https://www.mdpi.com/article/10.3390/ph16091206/s1, Table S1: Study selection.

## Appendix A. Study Characteristics

**Study**	**Methods**	**Population**	**Intervention**	**Outcomes**
Perić et al. (2020) [66]	Prospective case–control study	26 patients with acute post-viral rhinosinusitis	3×/day 20 mg oral EPs 7630 tablets for 10 days	Chemokine levels in nasal secretions, nasal symptoms and endoscopic findings
Perić et al. (2021) [65]	Randomized prospective study	78 patients with uncomplicated acute bacterial rhinosinusitis	During 10 days: 26 patients received 3 × 20 mg/day EPs 7630 tablets; 26 patients received 2 × 150 mg/day roxithromysin tablets	Chemokine levels in nasal secretion and clinical parameters were compared on day 0 and day 10
Goel et al. (2005) [81]	Randomized, double-blind, placebo controlled trial	150 volunteers, aged 18–65 years, with a history of two or more infections of the common cold in the previous year	At the onset of a cold, on day 1 eight doses of 5 mL Echinilin^TM^ (‘Factors R&D Technologies’, Burnaby, BC, Canada) were taken, and on the 6 consecutive days three doses/day	Self-assessed severity of cold symptoms during the 7 days
Dapas et al. (2014) [82]	Pilot study	10 healthy volunteers	10 mL Polinacea^®^ (Indena, Milano, Italy) syrup/day during 1 month	Expression levels of IL-2, IL-8, IL-6, and TNF-α in lympho-monocytes and plasma samples
Ritchie et al. (2011) [83]	Intervention study	30 healthy volunteers	Five days of 4 × 1 mL, followed by 3 days of 10 × 1 mL Echinaforce^®^	Elastase, IL-1β, IL-6, IL-8, IL-10, IL-12 MCP-1, TNF-α, and IFN-γ in daily taken blood samples
Kim et al. (2002) [84]	Preliminary, randomized, double-blind, placebo-controlled trial	48 healthy female volunteers, aged 22–51 years	Daily, a combination of *E. purpurea* and *E. angustifolia* (E. purpurea whole herb extract 4% (908 mg/day), *E. purpurea* whole herb (464 mg/day) or *E. angustifolia* root (36 mg/day) during 4 weeks	Complement properdin, white blood cell count, neutrophils, lymphocytes, monocytes, quality of life assessment
Sheeja et al. (2006) [46]	Animal study	12 BALB/c mice	5 doses of 10 mg methanolic extract of *A. paniculata* for 5 days	PMA-induced superoxide and nitric oxide formation
Wang et al. (2010) [42]	Animal study	12 BALB/c mice	Intraperitoneal administration of 1 mg/kg/day andrographolide, during 7 days	Antibodies and IL-4-producing splenocytes
Mishra et al. (2013) [34]	In vitro	*E. coli*, *P. aeruginosa*, *M. tuberculosis*, *S. Aureus*, MRSA, and methicillin-resistant *E. faecalis*	Agar well diffusion with *A. paniculata*	MIC
Abubacker and Vasantha (2010) [36]	In vitro	*E. coli*, *K. pneumoniae*, *P. vulgaris*, and *S. pneumoniae*	Disc diffusion assay with *A. paniculata*	Inhibition zone
Rajalakshmi and Cathrine (2016) [33]	In vitro	*E. coli*, *S. aureus*, *B. subtilis*	Screening for phytochemical components of *A. paniculata* and disc diffusion assay	Inhibition zone
Xu et al. (2012) [37]	In vitro	*S. epidermidis*, *P. aeruginosa*, *B. subtilis*	Microtitre plate broth dilution with noriridoids from the roots of *A. paniculata*	MIC
Chandrasekaran et al. (2010) [44]	In vitro	J774A.1 murine macrophages	Effect of an extract of *A. paniculata* leaves on inflammatory response to LPS	Inhibition of NO, PGE_2_, IL-1β, and IL-6
Chandrasekaran et al. (2011) [43]	In vitro	J774A.1 macrophages	Effect of 7 phytoconstituents (andrographolide, neoandrographolide, isoandrographolide, andrograpanin, 14-deoxy-11,12-didehydroandrographolide, 7-O-methylwogonin, and skullcapflavone-I) isolated from *A. paniculata* on inflammatory mediators	NO, PGE2, IL-1β, IL-6, LTB_4_, TXB_2_, and histamine
Parichatikanond et al. (2010) [45]	In vitro	Ionophore A23 187-induced human platelets	The effect of diterpenoids from *A. paniculata* on the production of inflammatory cytokines and COX activities	COX-2 activity, TNF-α, IL-6, IL-1β, and IL-10, gene expression of cytokines and cytokine receptors
Liu et al. (2007) [41]	In vitro	L-929 cells and bone-marrow-derived macrophages from BALB-c mice	The effect of andrograpanin from *A. paniculata* on overproduction of NO, pro-inflammatory cytokines, and involved pathways	NO, TNF-, IL-6, and IL-12p70
Kumar et al. (2004) [40]	In vitro	Peripheral blood lymphocytes of healthy volunteers	Different fractions of methanolic extract of *A. paniculata* were screened for immunostimulatory effects	IL-2
Chen et al. (2009) [38]	Animal and in vitro study	BALB/c mice	Mice were infected with avian influenza A/chicken/Guang-dong/96 (H9N2) virus and A/duck/Guangdong/99 (H5N1) virus and different doses of *A. paniculata* were administered	LD50 of influenza virus in mice, lung virus titer, in vivo and in vitro antiviral evaluation, toxicity of *A. paniculata*
Conrad et al. (2007) [49]	In vitro	Human peripheral blood	The effect of EPs 7630 on the activity of human peripheral blood cells	Phagocytosis and oxidative burst
Janecki et al. (2011) [49]	In vitro	Human HEp-2 epithelial cells and group A-streptococci (GAS)	The effect of EPs 7630, the methanol and non-methanol fraction, on GAS adhesion to Hep-2 cells	Percentage of change in adhesion
Neugebauer et al. (2005) [57]	In vitro	Ciliated cell cultures of human epithelium	The effect of EPs 7630 on ciliary beat frequency	Percentage of change in ciliary beat frequency
Koch and Wohn (2007) [62]	In vitro	Human neutrophils and granulocytes	The effects of EPs 7630 in concentrations between 0.3 and 30 µg/mL after 5 h incubation on antibacterial peptide production	Antibacterial protein’s neutrophil peptides 1–3 and bactericidal/permeablity-increasing protein
Kayser et al. (2001) [59]	In vitro	*Leishmania donovani*, murine macrophages	Effects of EPs 7630 in in vitro models for intracellular infection with *Leishmania* parasites, an extracellular *Leishmania* growth assay, a fibroblast-virus protection assay (IFN activity), a fibroblast-lysis assay (TNF activity) and a biochemical assay for inorganic nitric oxides (iNO) were employed	TNF release, IFN induction, NO
Thäle et al. (2008) [60]	In vitro	Bone-marrow-derived mouse macrophages, *Listeria monocytogenes*	Effects of EPs 7630 on immunomodulators	NO, IL-1, IL-12, TNF-α, CD40, CD119
Trun et al. (2006) [64]	In vitro	Non-infected and *Leishmania*-infected RAW 264.7 cells	Effect of EPs 7630 on cytokine production	Gene expression of NO synthase and IL-1, IL-12, IL-18, TNF-α, IFN-α, and IFN-γ
Kolodziej and Kinderlen (2007) [69]	In vitro	Non-infected and *Leishmania*-infected murine macrophages	Antibacterial effects of EPs 7630 and their effects on non-specific immune functions	iNOS, IFN-α, IFN-γ, TNF-α, IL-1, IL-10, IL-12, and IL-18
Nöldner and Koch (2004) [55]	Animal study	NMRI mice	Effect of oral administration of a *P. sidoides* extract (100–400 mg/kg); 1 h later sickness behaviour was induced by intraperitoneal injection of LPS (400 μg/kg)	One hour later, behavioural effects were examined in the ‘light/dark box model’ by monitoring exploratory activity for 3 min
Nöldner and Schötz (2007) [56]	Animal study	NMRI mice	Effect of oral administration of a EPs 7630 extract (100–400 mg/kg) and subfractions; 1 h later sickness behaviour was induced by intraperitoneal injection of LPS (400 μg/kg)	Time spent in light compartment, number of changes
Walther et al. (2020) [50]	In vitro	MDCK and HeLa cells	Antiviral effects of *P. sidoides* against different types of rhinovirus and influenza virus	Cytotoxicity, viral plaque production, viral neuraminidase activity
Bao et al. (2015) [54]	Animal study	ICR mice SPF-class and guinea pigs	Antitussive, secretolytic, and anti-inflammatory effects of EPs 7630 were assessed in animal experiments following oral administration at human equivalent doses	Degree of tracheal and bronchial lesions, bronchosecretolytic (phenol red), concentrations of SOD and MDA in serum
Roth et al. (2019) [52]	In vitro	Human bronchial cells from patients with severe asthma (*n* = 6), moderate COPD (*n* = 6) and non-diseased controls (*n* = 6)	The effect of EPs 7630 on human bronchial cells was assessed with western blot, immunofluorescence, and polymerase chain reaction	Protein expression, cell survival
Roth et al. (2021) [53]	In vitro	Human bronchial epithelial cells	The effect of EPs 7630 on human bronchial cells was assessed with western blot and immunofluorescence	Protein expression, intracellular signalling, antiviral effect
Witte et al. (2015) [63]	In vitro	Human monocytes (PBMCs), isolated from healthy donors	The effects of EPs 7630 on human monocytes was assessed by ELISA, western blot, and flow cytometry analyses	TNF-α, IL-6, and IL-10
Witte et al. (2020) [61]	In vitro	PBMCs	The effects of EPs 7630 on antimicrobial airway defense through monocytes	IL-22, IL-17, IFN-γ, IL-1, IL-23
Vimalanathan et al. (2017) [70]	In vitro	MDCK and BEAS-2B (human) cells. Influenza virus A and *H. influenzae* and *S. aureus*	Effect of an *E. purpurea* extract (Echinaforce^TM^) on H3N2-induced adhesion of live *H. influenzae* and *S. aureus*	Cytotoxicity, bacterial adhesion, expression of ICAM-1, fibronectin, PAFr, TLR4 and NFkB p65, IL-6 and IL-8
Fonseca et al. (2014) [80]	In vitro	Human Jurkat T-cells	Effect of different concentrations of *E. purpurea* extract (0, 10, 25, 100 and 250 μg/mL) on human T-cells was assessed	IL-2, IFN-γ, CD25 expression
Gertsch et al. (2004) [85]	In vitro	Human monocytes/macrophages	*Echinacea* alkylamides from Echinaforce^TM^	TNF-α protein and mRNA, cAMP, p38/MAPK, and JNK signaling, as well as NF-jB and ATF-2/CREB-1
Classen et al. (2006) [86]	In vitro	Mouse spleen cell cultures and mouse macrophages	Effects of different arabinogalactan-proteins from *E. paalida* were assessed	Proliferation, IgM production, induction of IL-6 and nitrite production
Keyhanmanesch et al. (2015) [93]	Animal study	Dunkin–Hartley guinea pigs	Intraperitoneal injection of different doses of α-hederin	IL-4, IFN-γ, and IL-17 levels in blood and histopathological evaluation of lungs and trachea
Sieben et al. (2009) [97]	In vitro	Human airway smooth muscle cells (HASM)	Cells were pre-treated with α-hederin, hederacoside, and hederagenin	β2 adrenergic receptor density, intracellular cAMP
Gepdiremen et al. (2005) [99]	Animal study	Wistar albino rats	Anti-inflammatory potential of a-hederin (monodesmoside) and hederasaponin-C from *H. helix* was assessed in carrageenan-induced acute paw edema in rats	Edema rate percentage
Wolf et al. (2011) [95]	In vitro	Bovine tracheal smooth muscle strips	Effects of the three main saponins of ivy, α-hederin, hederacoside C, and hederagenin, on the contraction and relaxation behaviour of isolated bovine tracheal smooth muscle strips by isometric tension measurements	Histamine-, methacholine-, and isoprenaline-induced relaxation of precontracted muscle strips
Hocauglu et al. (2012) [94]	Animal study	BALB/c mice	Mice were challenged with ovalbumin after which they received either a placebo, *H. helix* extract, or dexamethasone.	Goblet cell numbers, thickness of basement membrane, epithelium, and subepithelial smooth muscle layers
Saadat et al. (2015) [92]	Animal study	Dunkin–Hartley guinea pigs	Guinea pigs were sensitized with ovalbumin, after which a low (0.3 mg/kg) or high (3.0 mk/kg) dose of α-hederin was given	Tracheal response to methacholine, histamine and ovalbumin, white blood cell count
Shulte-Michels et al. (2016) [90]	In vitro	HEK293 cells	To assess the effect of *H. helix* extract on protein kinase A and G protein-coupled receptor kinases phosphorylation of β2 adrenergic receptors, an in-cell western was performed	Protein kinase A-mediated phosphorylation, G coupled receptor kinases phosphorylation
Greunke et al. (2015) [96]	In vitro	Human airway smooth muscle cells	The effect of an ivy leaf extract (EA 575) on human airway smooth muscle cells was assessed using live cell imaging, fluorescence correlation spectroscopy, and a cAMP assay	cAMP, internalization and binding of β-adrenergic receptors
Orhan et al. (2012) [88]	In vitro	*S. pneumonia*, *S. pyogenes*, *S. aureus*, *S. epidermidis*, *M. tuberculosis*, *K. pneumoniae*, *H. influenzae*, *P. auruginosa*, *A. baumannii*	Antibacterial effects of *H. helix* were assessed	DPPH inhibition, MIC
Schulte-Michels et al. (2019) [91]	In vitro	Mouse macrophages (J774.2), human embryonic kidney cells (HEK 293), acute monocytic leukemia cells (THP-1), and human lung epithelium-derived cells (A549)	Effect of EA 575 on transcriptional activity of NFkB was assessed	NFkB, IkBa and RelA, TNF-α
Schulte-Michels et al. (2016) [90]	In vitro	Murine macrophages (J774.2)	Different concentrations of EA 575 were tested for their effect on IL-6 release in macrophages	IL-6
Ünsal et al. (2010) [89]	Field study and in vitro study	*S. aureus*, *S. epidermidis*, *E. coli*, *K. pneumoniae*	During a three-month field study, 64 plant samples of plants that are traditionally used in common infections were collected and tested for antimicrobial activity	MIC
Acs et al. (2016) [108]	In vitro	Methicillin-resistant *Staphylococcus aureus* and the antibiotic-resistant *Pseudomonas aeruginosa*	With a tube dilution test, antimicrobial activity of thyme was assessed	MIC
Acs et al. (2018) [113]	In vitro	*S. pneumoniae*, *S. mutans*, *S. pyogenes*, *H. influenzae*, *H. parainfluenzae*, and *M. catarrhalis*	With broth microdilution test and vapor phase test, antimicrobial activity of thyme was assessed	MIC and MBC
Al-Bayati et al. (2008) [112]	In vitro	*S. aureus*, *B*. *cereus*, *E. coli*, *P. vulgaris*, *P. mirabilis*, *S. typhi*, *S. typhimurium*, *K. pneumoniae*, and *P.* *aeruginosa*	With microwell dilution method, the antimicrobial activity of *T. vulgaris* was assessed	MIC
Csikos et al. (2020) [117]	Animal study	C57BL/6J mice	Acute airway inflammation was induced in mice, essential oil of thyme was inhaled by one group of the animals	Airway function, hyperresponsiveness, and myeloperoxidase activity
Dahiya and Purkayastha (2012) [101]	In vitro	MDR clinical isolates of *S. aureus* (3 isolates), *S. aureus* (MRSA), *Escherichia coli* (3 isolates), *K. pneumoniae* (2 isolates), and *P.* *mirabilis*	With the agar well diffusion method, the antimicrobial activity of thyme was assessed	MIC
Fabio et al. (2007) [107]	In vitro	*S. pyogenes*, *S. agalactiae*, *S.* *Pneumoniae*, *K. pneumoniae*, *H. influenzae*, *S. aureus*, *S. maltophilia*	With the Kirby–Bauer paper method	Cytotoxicity, MIC, and MBC
Hammer et al. (1999) [111]	In vitro	*Aer. Sobria*, *Candida albica*, *E. faecalis*, *E.* *coli*, *K. pneumoniae*, *P. aeruginosa*, *S. enterica*, *S. marcescens*, and *S. aureu*	With the agar dilution method and the broth dilution method, the antimicrobial activity of essential oil of *T. vulgaris* was assessed	MIC and MCC
Hoferl et al. (2009) [114]	In vitro	*S. aureus*, *E. coli*, *P. aeruginosa*, *P. vulgaris*, *K. pneumoniae,* and *S. sp.*	With agar diffusion disc method and agar dilution method, the antimicrobial activity of thyme was assessed	MIC and inhibition zone (IZ)
Inouye et al. (2001) [103]	In vitro	*H. influenzae*, *S. aureus*, *S. pneumoniae*, *E. coli*, and *S. pyogenes*	A minimum inhibitory dose was established to assess the antimicrobial activity of thyme	Minimum inhibitory dose (MID)
Iseppe et al. (2020) [104]	In vitro	*E. coli*, *K. pneumoniae*, *P. aeruginosa*	With agar diffusion disc method, the antimicrobial activity of essential oil of thyme was assessed	MIC
Javed et al. (2018) [100]	In vitro	*S. aureus*, *P. aeruginosa*, *E. coli*, *P. vulgaris*, *K. pneumoniae*	With the disk diffusion method, the antibacterial activity of *T. vulgaris* was assessed	MIC
Kwiatkowski et al. (2018) [109]	In vitro	*K. pneumoniae*	With broth microdilution method, the antimicrobial activity of essential oil of *T. vulgaris* was assessed	MIC, MBC
Lelesius et al. (2019) [116]	In vitro	Avian infectious bronchitis virus (IBV)/Vero Cells	Antiviral effects of *T. vulgaris* were assessed	Cytotoxicity, antiviral effect against IBV, and prior to infection in Vero cells
Man et al. (2016) [106]	In vitro	*S. aureus*, *E. faecalis*, *E. coli*, *K. pneumoniae*, *P. aeruginosa*	Antibacterial effects of *T. vulgaris* were assessed	MIC, MBC
Marchese et al. (2016) [32]	Review	n.a.	Antibacterial and antifungal activities of thymol are researched in the literature	Chemistry, bioavailability, safety assessment, biological activity, antibacterial, and antifungal activity
Mohamed et al. (2019) [115]	In vitro	*K. pneumoniae*	Assessment of antibacterial effects of thymol, also in combination with ampicillin with disc diffusion assay and checkerboard assay	MIC, IZ
Mohammed et al. (2018) [105]	In vitro	*K. pneumoniae*	Assessment of antibacterial effects of essential oil of thyme, also in combination with ciprofloxacin with disc diffusion assay and checkerboard assay	MIC, MBC
Seibel et al. (2015) [120]	Animal study	Wistar rats	Bronchoalveolitis was induced in rats, which were then given different doses of Bronchipret (combination of ivy/primrose/thyme)	Granulocyte infiltration, effects on leukocytes, goblet cells in bronchial tissue, and leukotriene production and 5-LO enzyme activity
Van Vuuren et al. (2008) [121]	In vitro	*S. aureus*, *K. pnuemoniae*, *C. albicans*	Essential oil of *T. vulgaris* was assessed for antimicrobial activity, also in combination with conventional antimicrobials	MIC
Vazquez-Ucha et al. (2020) [110]	In vitro	*A. baumannii* and *K. pneumoniae*	Combined disk diffusion test and checkerboard assays were performed with the essential oil of *T. zygis* in combination with colistin	MIC
Wani et al. (2021) [102]	Review	n.a.	Literature review of antiviral potential of essential oils (including thyme)	Chemical composition, antiviral effects
Samuel and Priyadarshoni (2019) [75]	Review	n.a.	Literature review on biologic effects of *Echinacea* species	Active components, pharmacological actions, medicinal effects
Rondanelli et al. (2018) [76]	Review	n.a.	Literature review on the use of *Echinacea* in self-care for common colds	Effects on cellular immunity and adaptive immunity
Roxas and Jurenka (2007) [79]	Review	n.a.	Literature review on the use of *Echinacea* in treatment of the common cold	Incidence, signs and symptoms, potential complications, conventional treatment, alternative treatments
Barnes et al. (2005) [72]	Review	n.a.	Literature review on *E. angustifolia*, *E. pallida*, and *E. purpurea*	Chemistry, pharmacology, and clinical properties
Islam and Carter (2005) [87]	Review	n.a.	Literature review on the use of *Echinacea* in upper respiratory tract infections	Qualitative and quantitative properties, mechanism of action, clinical trials, and its effect on the immune system
Barret (2003) [73]	Review	n.a.	Literature review on the medicinal properties of *Echinacea*	Pharmacology, safety, contraindications, risk of adverse events, phytochemistry
Bash et al. (2005) [74]	Review	n.a.	Natural standard review on *E. angustifolia*, *E. pallida*, and *E. purpurea*	Toxicology, dosing, interactions, mechanism of action
Borchers et al. (2000) [77]	Review	n.a.	Literature review on *Echinacea* species	Anti-inflammatory activities, *Echinacea* species
Moyo and Van Staden (2014) [26]	Review	n.a.	Literature review on the uses and mechanism of action of *P. sidoides*	Phytochemistry, pharmacological properties, commercial potential, biotechnology application
Kolodziej et al. (2011) [67]	Review	n.a.	Literature review on the antimicrobial, antiviral, and immunomodulatory activities of *P. sidoides*	Respiratory tract related antibacterial activity, effect on mucociliary system, antiviral effects, immunomodulatory effects
Brendler et al. (2008) [68]	Review	n.a.	Literature review on pre-clinical and clinical studies of *P. sidoides*	Historical and commercial perspectives, scientific perspectives
Hossain et al. (2021) [35]	Review	n.a.	Literature review on *A. paniculata*	Phytochemistry, antimicrobial pharmacology, and clinical safety and efficacy
Jiang et al. (2021) [39]	Review	n.a.	Literature review on *A. paniculata* and andrographolide	Ethnopharmacology, phytochemicals, antiviral properties, toxicology
Kumar et al. (2021) [24]	Review	n.a.	Literature review on *A. paniculata*	Traditional uses, phytochemistry, pharmacology, and quality control
Okhuarobo et al. (2014) [47]	Review	n.a.	Literature review on *A. paniculata*	Phytochemistry and pharmacology

## Appendix B. Search Terms

### Appendix B.1. Search 1: Reviews on All Herbs

(Bronchiolit* OR bronchit* OR bronchopneumon* OR “Common Cold” OR Cough OR Croup OR laryngit* OR “Parainfluenza Virus” OR laryngotracheobronchit* OR nasopharyngit* OR nasosinusit* OR pharyngit* OR Pleurisy OR pneumon* OR pleuropneumon* OR Pseudocroup OR “respiratory infection*” OR “Respiratory Tract Infections” OR “Respiratory Tract Infection*” OR Rhinit* OR rhinopharyngit* OR rhinosinusit* OR rhinorrh* OR sinusit* OR Sneez* OR “blocked nose” OR “pain* throat*” OR “sore throat*” OR “strep throat” OR “Tonsillitis” OR tonsillit* OR “Scarlet Fever” OR “scarlet fever” OR tracheobronchit* OR URTI) AND (“Andrographis paniculat*” OR Chuan Xin Lian OR Creat* OR “green chiretta” OR nilavembu OR kariya* OR sanshin* OR “ch’onsimyon” OR Andrographol* OR “Pelargonium sidoides” OR “African geranium*” OR “black geranium*” OR “calvary bossia” OR “cape perlargonium” OR Umckaloab* OR Rabassamin OR zucol OR “Echinacea” OR Echinacea OR echinacoside OR “Primula” OR Primula OR “P. veris” OR “P. elatior” OR “Thymus Plant” OR Thyme OR “T. vulgaris” OR “T. zygis” OR Umckaloabo OR Ivy OR “Hedera” OR “Hedera helix” OR Primrose OR Thymus OR “cow slip”) AND (“Anti-Bacterial Agent*” OR “Antibacterial agent*” OR “Anti-Infective Agent*” OR “AntiInfective Agent*” OR “Antimicrobial agent*” OR “Anti-microbial agent*” OR “Antiviral Agent*” OR “Anti-viral Agent*” OR Resilien* OR immun* OR “ antipyretic* OR febrifug* OR Bioactivity OR “mechanism of action” OR Mechanistic OR “working mechanism*”) AND (“systematic review” OR meta-analysis OR review)

### Appendix B.2. Search 2: Mechanistic studies on Ivy, Primrose, and Thyme

(Bronchiolit* OR bronchit* OR bronchopneumon* OR “Common Cold” OR Cough OR Croup OR laryngit* OR “Parainfluenza Virus” OR laryngotracheobronchit* OR nasopharyngit* OR nasosinusit* OR pharyngit* OR Pleurisy OR pneumon* OR pleuropneumon* OR Pseudocroup OR “respiratory infection*” OR “Respiratory Tract Infections” OR “Respiratory Tract Infection*” OR Rhinit* OR rhinopharyngit* OR rhinosinusit* OR rhinorrh* OR sinusit* OR Sneez* OR “blocked nose” OR “pain* throat*” OR “sore throat*” OR “strep throat” OR “Tonsillitis” OR tonsillit* OR “Scarlet Fever” OR “scarlet fever” OR tracheobronchit* OR URTI) AND (“Primula” OR Primula OR “P. veris” OR “P. elatior” OR “Thymus Plant” OR Thyme OR “T. vulgaris” OR “T. zygis” OR Umckaloabo OR Ivy OR “Hedera” OR “Hedera helix” OR Primrose OR Thymus OR “cow slip”) AND (“Anti-Bacterial Agent*” OR “Antibacterial agent*” OR “Anti-Infective Agent*” OR “AntiInfective Agent*” OR “Antimicrobial agent*” OR “Anti-microbial agent*” OR “Antiviral Agent*” OR “Anti-viral Agent*” OR Resilien* OR immun* OR “ antipyretic* OR febrifug* OR Bioactivity OR “mechanism of action” OR Mechanistic OR “working mechanism*”)

### Appendix B.3. Search 3: Recent Studies on Pelargonium

(“Pelargonium sidoides” OR “African geranium*” OR “black geranium*” OR “calvary bossia” OR “cape perlargonium” OR Umckaloab* OR Rabassamin OR zucol OR Umckaloabo) AND (“Anti-Bacterial Agent*” OR “Antibacterial agent*” OR “Anti-Infective Agent*” OR “AntiInfective Agent*” OR “Antimicrobial agent*” OR “Anti-microbial agent*” OR “Antiviral Agent*” OR “Anti-viral Agent*” OR Resilien* OR immun* OR “ antipyretic* OR febrifug* OR Bioactivity OR “mechanism of action” OR Mechanistic OR “working mechanism*”) AND(“pharmacological effect*” OR “therapeutic effect*” “molecular mechanism*” OR “treatment efficacy” OR “treatment outcome*” OR “outcome* assessment*” OR “therapeutic equivalen*”)
